# Development of UROGRAFT: A Bladder Acellular Matrix-Based Composite for Advanced Cystoplasty, Highlighting the Role of Graft Shape and Composition

**DOI:** 10.1021/acsbiomaterials.5c00700

**Published:** 2025-07-16

**Authors:** Marta Pokrywczynska, Zuzanna Fekner, Daria Balcerczyk, Tomasz Kloskowski, Marta Rasmus, Damian Kasinski, Michal Stopel, Marta Szulc, Jan Adamowicz, Arkadiusz Jundzill

**Affiliations:** † Chair of Urology and Andrology, Department of Regenerative Medicine, 49604 Nicolaus Copernicus University in Torun, Medical College in Bydgoszcz, Sklodowskiej-Curie M. Street, Bydgoszcz 85-094, Poland; ‡ Department of Mechanical Engineering, 111405 Bydgoszcz University of Science and Technology, Al. Prof. S. Kaliskiego 7 Street, Bydgoszcz 85-796, Poland; § Faculty of Chemistry, Department of Biomaterials and Cosmetics Chemistry, 49577 Nicolaus Copernicus University in Torun, Gagarina 7 Street, Torun 87-100, Poland

**Keywords:** augmentation cystoplasty, urinary bladder regeneration, tissue engineering, graft, mesenchymal stromal cells, bladder acellular matrix

## Abstract

Urinary bladder augmentation with gastrointestinal segments, despite many complications, remains a gold standard treatment of low-capacity, poorly compliant, or refractory overactive urinary bladder. In this study, we developed the UROGRAFT, a new bladder acellular matrix–collagen–cellulose (BAM–CC) composite for urinary bladder augmentation. The study presents the step-by-step development process of UROGRAFT, including the selection of an optimal decellularization protocol and cross-linking method to ensure optimal biomaterial properties. Histological and biochemical analyses demonstrated that the combined protocol of Triton X-100 and sodium dodecyl sulfate (SDS) was the most effective, completely removing cellular components while preserving the extracellular matrix (ECM). DNA quantification confirmed a significant reduction in residual genetic material, ensuring a low immunogenic profile. Scanning electron microscopy (SEM) confirmed high porosity and well-preserved collagen fibers. To reduce porosity and permeability, BAM was cross-linked with collagen type I and dialdehyde carboxymethyl cellulose, optimizing scaffold performance. Biocompatibility tests confirmed the absence of toxicity, tissue reactions, acute systemic toxicity, and mutagenic effects. Based on computational modeling, verified by implantation trials, a unique three-armed graft shape resembling lily petals was developed. A preclinical study in porcine models demonstrated that UROGRAFT is highly biocompatible, well-tolerated, and safe for urinary bladder augmentation. Composite BAM–CC scaffolds provide an appropriate environment for adipose derived mesenchymal stromal cells (AD-MSCs) growth; therefore, the UROGRAFT can be used in the future as an acellular graft (biomedical device) or a cell-seeded tissue-engineered product (combined ATMP-biomedical device). UROGRAFT developed in this study is a promising new product with the potential to be used in augmentation cystoplasty, offering a safe and effective alternative to gastrointestinal segments.

## Graphical abstract

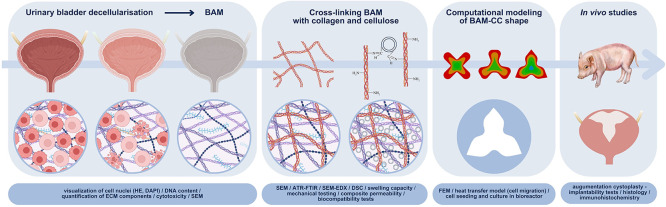

## Introduction

Urinary bladder reconstruction is often required due to various congenital and acquired conditions that compromise bladder function. In children, congenital abnormalities such as bladder exstrophy, myelomeningocele, and posterior urethral valves can result in bladders that are both low in capacity and poorly compliant. In adults, bladder dysfunction may arise from neurogenic causes (e.g., spinal cord injury, multiple sclerosis, and myelodysplasia) as well as non-neurogenic causes such as detrusor instability, chronic inflammatory conditions (e.g., interstitial cystitis, tuberculosis, and schistosomiasis), postradiation cystitis, and iatrogenic injuries.^1^

Augmentation cystoplasty remains a crucial intervention for patients with refractory overactive, low-capacity, or poorly compliant bladders when conservative therapies fail.^2^ Traditionally, the use of autologous gastrointestinal tissues has been considered the gold standard; however, these approaches are associated with significant complications—including bowel dysfunction, metabolic imbalances, urolithiasis, malignant transformations, and even life-threatening bladder perforation.^3–5^ Consequently, there is a pressing need for innovative therapeutic alternatives that avoid these adverse outcomes.

Various strategies have been studied in order to find the most appropriate scaffold for urinary bladder augmentation including: biological scaffolds derived from natural tissues like bladder acellular matrix (BAM),^6,7^ small intestinal submucosa (SIS)^8,9^ or amniotic membrane;^10^ natural polymers like collagen,^11^ chitosan,^12^ silk;^13^ synthetic polymers like poly­(lactic acid), poly­(glycolic acid), poly­(lactic-*co*-glycolic acid) and their composites.^14^ The overwhelming number of these studies concern small animal models with no clinical translation.

Biological scaffolds based on extracellular matrix (ECM) are of particular interest because they create a favorable pro-regenerative microenvironment. ECM provides not only physical support (a native framework for cell adhesion at the site of tissue defect that allows local cells to migrate, proliferate, and differentiate) but also a reservoir for growth factors, morphogens, and other bioactive molecules that serve as an instructive template for tissue regeneration.^15,16^

Previously, in our preclinical studies on the porcine model, we found that bladder acellular matrix-based scaffolds possess very good biocompatibility, biodegradation profile, and mechanical properties. However, insufficient urine barrier increases local inflammation and tissue fibrosis in the central graft region, which are major challenges in transferring BAM-based tissue-engineered grafts into routine clinical practice in urology.^17,18^ We found that the urinary bladder environment is extremely unfavorable for regeneration. Urine is highly cytotoxic to implanted cells; consequently, many cells die immediately following implantation, not as a result of lack of graft angiogenesis but under the cytotoxic influence of urine.^17^ This highlights a critical research gap: the need for a tissue-engineered urinary bladder substitute that not only supports cellular regeneration and mimics the mechanical properties of native tissue but also effectively resists urine permeation and minimizes fibrotic responses.

To address this gap, we developed UROGRAFT, a novel composite scaffold for urinary bladder augmentation. UROGRAFT is synthesized by cross-linking bladder acellular matrix with collagen type I and dialdehyde carboxymethyl cellulose (BAM–CC). Such modification was intended to reduce scaffold porosity and urine permeability. Additionally, the scaffold’s unique design promotes recellularization by native bladder cells and provides mechanical properties suitable for both bladder filling and emptying. In this study, we detail the step-by-step development of UROGRAFT, including its synthesis, comprehensive characterization, and evaluation both on small and large animal models, with a focus on biocompatibility and the potential for effective bladder tissue regeneration (Figure 1).

**Figure 1:**
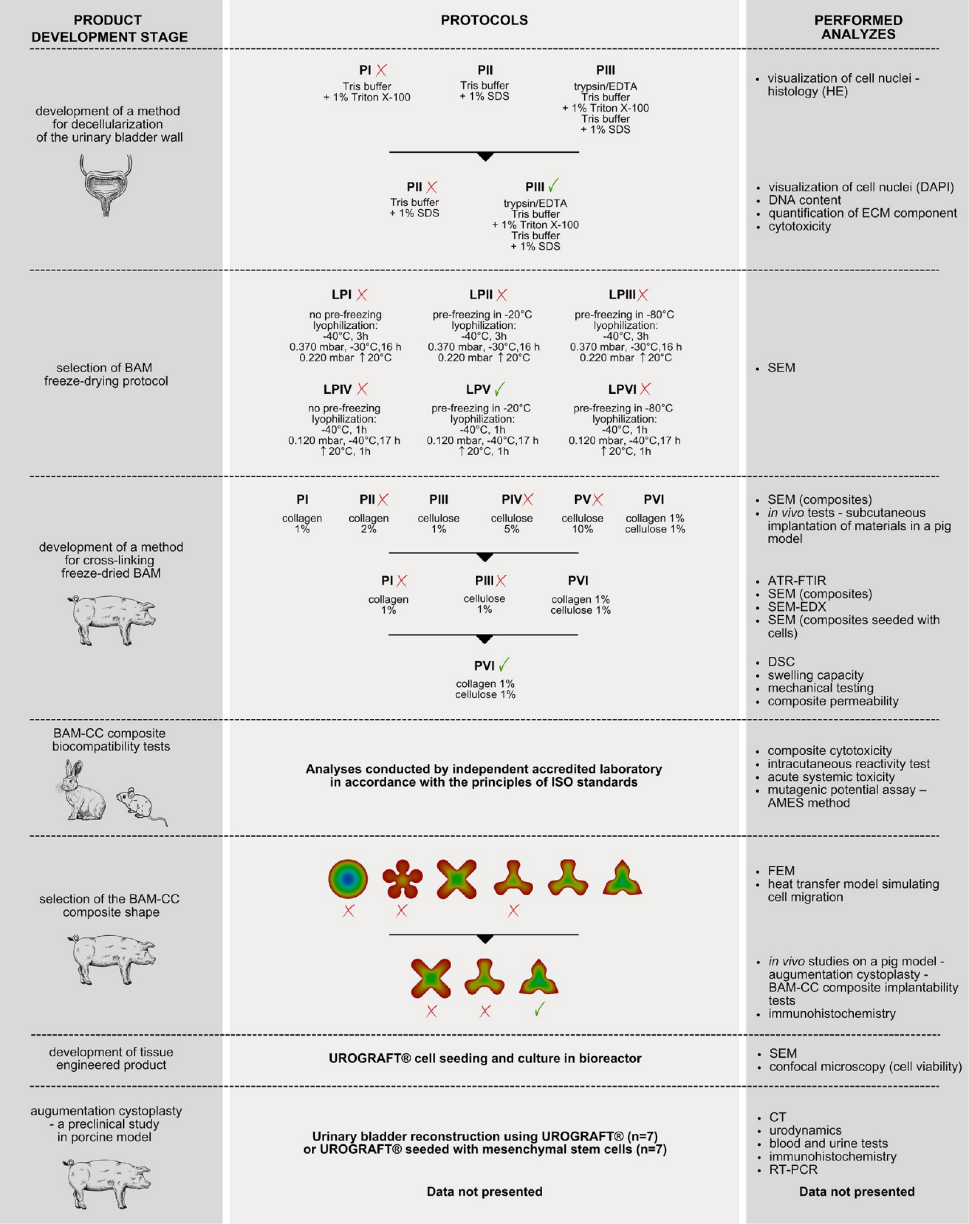
A general outline of the course of experiments, specifying the main stages of product development, protocols used at individual stages, and performed analyses; P – protocol, LP – lyophilization protocol.

By integrating recent advancements in scaffold design and addressing previous limitations in urinary bladder reconstruction, our research aims to pave the way for safer and more effective clinical applications in reconstructive urology.

## Materials and Methods

### Ethics Statement

The experimental protocol was approved by the Nicolaus Copernicus University Bioethics Committee (no. 705/2018) and the Local Ethical Committee for Animal Experiments (no. 46/2018).

### Decellularization of Porcine Urinary Bladder

A dynamic decellularization protocol representing the most appropriate approach for removing cells from the whole organs was used for the decellularization of porcine urinary bladders.

### Automatic System for Dynamic Bladder Decellularization

In order to conduct this process, a homemade dynamic decellularization system was created, comprising a plastic box and an aquarium pump, thereby ensuring a constant flow of decellularizing solutions through the urinary bladder tissues. Following the initial trials and the analysis of the results obtained, the decision was made to proceed with the development of a fully automated tissue decellularization system. The final, fully automated system was manufactured by Zellwerk GmbH (Germany), ensuring high precision and repeatability in the process. This system significantly increased the efficiency and effectiveness of the decellularization process.

Urinary bladders were placed within the automated decellularization system (Zellwerk, Germany), where they were connected in such a manner as to ensure a continuous flow of decellularizing solutions. The solution was introduced into the bladder via tubing-stabilized ureters while the fluid flowed out through the urethral outlet, which was stabilized with a 12 mm diameter ring to ensure even and effective flushing of all anatomical structures of the bladder. This continuous flow of decellularization solutions ensured uniform and effective removal of cells and cellular debris from bladder tissues (Figure 2A).

**Figure 2:**
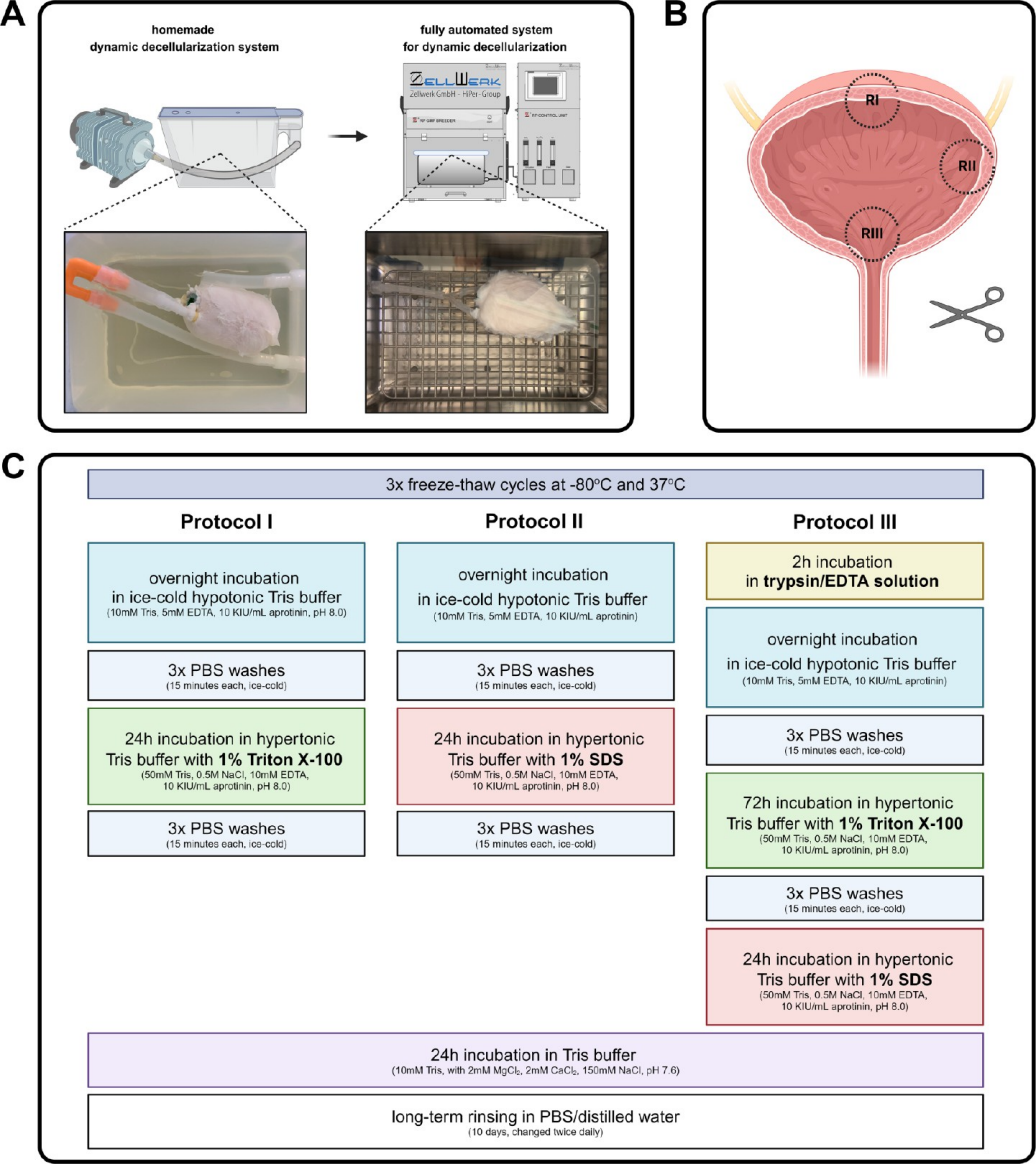
(A) Homemade dynamic decellularization system and fully automated system for dynamic decellularization (Zellwerk, Germany); (B) distribution of regions I–III (RI–III) of samples harvested for histological and fluorescence analyses; (C) overview of protocols I–III used to manufacture bladder acellular matrices (BAMs).

Porcine urinary bladders (*n* = 24) were obtained from domestic pigs (100–120 kg) at the planned market slaughter. The bladders were transported in ice-cold transport media with antibiotics (DMEM/F-12; Corning, USA) to the laboratory, where the fat tissues and other adjacent tissues were removed entirely. The bladders were rinsed with PBS (Corning, USA), filled with PBS/EDTA 0.01%, and frozen at −80 °C.

Decellularization was carried out according to the protocol developed by Yang et al.^19^ with some modifications (protocols I–III). Before the main part of decellularization, the bladders were subjected to three freeze–thaw cycles at −80 and 37 °C to disrupt cell structures. Three different protocols for automated decellularization of the whole urinary bladder have been tested using trypsin/EDTA to remove urothelium (III), hypotonic buffer, Triton X-100 (I, III) or/and SDS (II, III) in a hypertonic buffer to remove membranous and cytoplasmic materials (Figure 2C).

### Protocol I

The bladders (*n* = 4) were connected to the automated decellularization system. Next, the bladders were incubated overnight in ice-cold hypotonic Tris buffer (10 mM Tris, pH 8.0, VWR International, USA) containing 5 mM ethylenediaminetetraacetic acid (EDTA; VWR International, USA) and 10 KIU/mL aprotinin (Sigma-Aldrich, Germany), and then the tissues were washed thrice with ice-cold PBS for 15 min. The bladder was then incubated for 24 h at room temperature (RT) in hypertonic Tris buffer (50 mM Tris, 0.5 M NaCl, 10 mM EDTA, and 10 KIU/mL aprotinin, pH 8.0) containing 1.0% Triton X-100 (Sigma-Aldrich, Germany).

### Protocol II

Bladder decellularization performed in protocol II (*n* = 5) was similar to that in protocol I, except that the hypertonic Tris buffer contained 1.0% SDS instead of Triton X-100.

### Protocol III

In protocol III, the bladders (*n* = 12) were connected to the automated decellularization system. First, the bladders were incubated in the 0.25% trypsin/EDTA solution for 2 h at room temperature (RT) and then washed thrice with ice-cold PBS for 15 min. Then, the bladders were incubated overnight in an ice-cold hypotonic Tris buffer (10 mM Tris, pH 8.0) containing 5 mM ethylenediaminetetraacetic acid (EDTA) and 10 KIU/mL aprotinin. After that, the bladders were washed thrice with ice-cold PBS for 15 min, incubated for 72 h at room temperature (RT) in hypertonic Tris buffer (50 mM Tris, 0.5 M NaCl, 10 mM EDTA, and 10 KIU/mL aprotinin, pH 8.0) containing 1.0% Triton X-100, and then washed again thrice with ice-cold PBS for 15 min. Finally, the bladders were incubated for 24 h in hypertonic Tris buffer (50 mM Tris, 0.5 M NaCl, and 10 mM EDTA, pH 8.0) containing 1.0% SDS (Sigma-Aldrich, Germany).

The obtained bladder acellular matrices (BAMs) in protocol I, II, and III were washed thrice with ice-cold PBS for 15 min and then subjected to 10 mM Tris buffer (pH 7.6) containing 2 mM MgCl_2_, 2 mM CaCl_2_, and 150 mM NaCl for 24 h at RT. Next, the obtained BAM was washed thrice with ice-cold PBS, followed by rinsing in distilled water/PBS for 10 days (2 changes daily) at RT. BAM rinsing was optimized to remove cellular debris and detergent residues.

### Histological and Fluorescence Analysis

Due to the observed differences in the thickness of the BAM manufactured, samples from three regions of decellularized bladders were analyzed: the apex (RI), the body (RII), and the area near the base of the bladder (RIII) (Figure 2B). This approach allowed a detailed assessment of the structure and quality of the tissue and confirmed the feasibility of using each fragment for graft manufacture. Fragments measuring 1 cm × 1 cm were taken from three regions of native and decellularized bladders. Samples were fixed in 10% phosphate-buffered formalin for 24 h, dehydrated, embedded in paraffin, and sliced into 4 μm-thick sections. Slides were stained with hematoxylin and eosin (H&E) and 4′,6-diamidino-2-phenylindole (DAPI), allowing visualization of cell nuclei and ECM structures. Decellularization efficiency was quantified by comparing the number of nuclei in decellularized and native bladder tissue samples. Analysis was performed on fields of 0.07 mm^2^ (H&E) and 0.04 mm^2^ (DAPI) using the DM6000B microscope (Leica, Germany) at 40× magnification.

### Lyophilization

The lyophilization process was performed using the Alpha 2–4 LSCbasic lyophilizer (Martin Christ Gefriertrocknungsanlagen GmbH, Germany). Optimal lyophilization conditions were determined. For this purpose, three scaffold preparation methods and two lyophilization methods, resulting in a total of six lyophilization protocols (LP), were compared. BAM fragments (20 × 20 mm) were prepared by stretching to prevent curling and divided into three groups: without prefreezing, prefrozen at −20 °C, and prefrozen at −80 °C.

In LP I, samples were frozen at −40 °C for 3 h, primary drying at 0.370 mbar and −30 °C for 16 h, followed by secondary drying at 0.220 mbar and temperature raised to 20 °C. LP II additionally included prefreezing at −20 °C, and for LP III there was prefreezing at −80 °C. LP IV included freezing at −40 °C for 1 h, primary drying at 0.120 mbar and −40 °C for 17 h, and secondary drying with the temperature raised to 20 °C for 1 h. LP V additionally included prefreezing at −20 °C, and LP VI was prefreezing at −80 °C.

### DNA Content

The total genomic DNA from freeze-dried samples of 10 ± 1 mg of native porcine bladders and manufactured BAMs was extracted and qualified using the DNeasy Blood Tissue Kit (Qiagen, Germany), according to the manufacturer’s protocol. DNA concentrations were determined using a NanoDrop spectrophotometer (Thermo Fisher Scientific, USA) at an absorbance of 260 nm. The quantity of DNA in both native and decellularized tissues was expressed as mg DNA/mg of dry weight, based on a λDNA standard curve. This assessment evaluated the decellularization protocols’ effectiveness in removing genetic material from the tissue.

### Scanning Electron Microscopy

The BAMs ultrastructure was examined using a scanning electron microscope (Quanta 3D FEG). BAMs and native bladder samples were analyzed after lyophilization without any additional preparation. For cell-seeded biomaterials, samples were fixed for 24 h at 4 °C in 2.5% glutaraldehyde (Sigma-Aldrich, Germany) in 0.1 M phosphate-buffered saline, pH 7.4. Rinsing with PBS was followed by fixation in 1% osmium tetroxide (Sigma-Aldrich, Germany). Dehydration was carried out with a graded ethanol series; samples were dried to the critical point and coated with gold by sputtering. Finally, images were obtained using a scanning electron microscope (Quanta 3D FEG), allowing detailed surface morphology and ultrastructure observation.

### Quantification of ECM Component

The content of extracellular matrix proteins—collagen, elastin, laminin—in both the obtained BAMs and native bladders was assessed using the Collagen Assay Kit A (Sigma-Aldrich), the Fastin Elastin Assay (Biocolor, UK) and the ELISA Kit for Laminin (CloudClone, USA) according to the manufacturer’s recommended protocols. These assays quantify the protein content of the sample. The results were read on a Varioskan LUX Plate Reader (Thermo Scientific, USA).

### Isolation, Culture, and Characterization of Cells Used in Experiments

Mesenchymal stromal cells (MSCs) were isolated from porcine adipose tissue as described previously.^17^ Adipose derived-mesenchymal stromal cells (AD-MSCs) were cultured until the third passage in Dulbecco’s Modified Eagle Medium/Ham’s F12 (DMEM/Ham’s F12, Corning, USA) supplemented with 10% fetal bovine serum (FBS, Biowest, USA), 10 ng/mL basic fibroblast growth factor (b-FGF, Gibco, USA), 100 U/mL penicillin, 100 μg/mL streptomycin (HyClone, USA), and 5 μg/mL amphotericin B (Corning, USA). The cultures were maintained under standard conditions (37 °C, 5% CO_2_). Immunophenotype and multipotential character of AD-MSCs were confirmed by expression analysis of positive: CD29, CD90, CD44, and negative: CD11b, CD31, CD45 markers, and differentiation into osteogenic, chondrogenic, and adipogenic lineages, respectively (data not shown).

### Bladder Acellular Matrix Cytotoxicity

The cytotoxicity of BAM was evaluated according to ISO 10993–12:2012. For this purpose, the prepared lyophilized BAM fragments (0.1 g/mL) were incubated in a growth medium Dulbecco’s Modified Eagle Medium/Ham’s F12 (DMEM/Ham’s F12) (Corning, USA) supplemented with 10% fetal bovine serum (FBS) (Biowest, USA), 10 ng/mL basic fibroblast growth factor (b-FGF) (Gibco, USA), 100 U/mL penicillin and 100 μg/mL streptomycin (HyClone, USA), and 5 μg/mL amphotericin B (Corning, USA) at 37 °C for 24 h to prepare extracts. Four concentrations (100%, 50%, 25%, and 12.5%) were tested to assess potential dose–response effects. AD-MSCs were treated with 100%, 50%, 25%, and 12.5% BAM extracts or control medium for 24 h. The MTT assay determined cell viability; the resulting formazan crystals were dissolved in dimethyl sulfoxide (DMSO, POCH, Poland). Absorbance was measured at 570 nm (test wavelength) and 655 nm (reference wavelength) using a Varioskan LUX Plate Reader (Thermo Fisher Scientific, USA). Relative viability was calculated by comparing the absorbance in extract-treated cells with the control group.

### Manufacture of Cross-Linked BAM Scaffolds

In order to develop an effective method of BAM cross-linking to reduce the scaffold’s pore size and their permeability to urine, two polymers were compared: collagen type I (Symatese, France) and dialdehyde carboxymethyl cellulose (Gelita AG, Germany). Polymer solutions were incubated with BAM to obtain BAM-collagen (1% and 2%) and BAM-cellulose (1%, 5%, 10%) composites. The resulting composites were incubated at 4 °C for 12 h; then, samples were frozen and lyophilized. In addition, a double cross-linking method, BAM-collagen-cellulose, was also used. In stage I, BAM scaffolds were incubated with collagen solution (1%) for 24 h at 37 °C. In stage II, dialdehyde cellulose solution was added to the resulting composite and incubated for 48 h at 37 °C. Then, to increase the durability of the bonds between BAM and the polymers, a solution of sodium borohydride dissolved in ethanol was added to the obtained composites. The reaction took place for 3 h at 4 °C. Then, to mask the free groups, the composite was placed in 0.1 M glycine/PBS solution and left for 2 h at room temperature.

### Choosing the Right Method for BAM Cross-Linking

Numerous analyses were carried out in order to select a suitable method for BAM cross-linking, among them scanning electron microscope (SEM), attenuated total reflectance Fourier-transform infrared spectroscopy (ATR-FTIR), SEM-EDX, differential scanning calorimetry (DSC), swelling analysis and mechanical testing.

Scanning electron microscopy (SEM) was carried out to determine the degree of cross-linking of the BAM composites based on the number and size of pores formed. Scanning electron microscope (SEM) analysis with silver sputtering was performed at the Instrumental Analysis Laboratory of the Nicolaus Copernicus University in Torun. Scanning electron microscope (SEM) analysis was carried out for all composites tested: BAM–collagen (1% and 2%), BAM–cellulose (1%, 5%, 10%), and BAM–collagen (1%)–cellulose (1%).

Attenuated total reflectance Fourier-transform Infrared spectroscopy (ATR-FTIR) was carried out to determine BAM composites’ cross-linking degree. ATR-FTIR analysis was performed at the Instrumental Analysis Laboratory of the Nicolaus Copernicus University in Torun. ATR-FTIR analysis was carried out for composites tested: BAM–collagen (1%), BAM–cellulose (1%), and BAM–collagen (1%)–cellulose (1%).

SEM-EDX analysis was used to detail the elemental composition of the samples, with a particular focus on the presence of heavy metals, which is important for assessing the safety of the materials and potential environmental and health impacts. SEM-EDX analysis was performed at the Instrumental Analysis Laboratory of the Nicolaus Copernicus University in Torun. SEM-EDX analysis was carried out for composites tested: BAM–collagen (1%), BAM–cellulose (1%), and BAM–collagen (1%)–cellulose (1%).

Differential scanning calorimetry (DSC) analysis was carried out to determine the thermal stability of the composites tested. DSC analysis was performed at the Instrumental Analysis Laboratory of the Nicolaus Copernicus University in Torun. DSC analysis was carried out for composites tested: BAM and BAM–collagen (1%)–cellulose (1%).

Swelling analysis was conducted to determine how the composites tested react in an aqueous environment, reflecting their behavior under physiological conditions. Swelling analysis was carried out for composites tested: BAM and BAM–collagen (1%)–cellulose (1%). Swelling analysis was performed using pH 5 and 8 buffers and PBS. The analysis lasted 2 weeks and was carried out at 37 °C.

The mechanical properties of the tested biocomposites were also compared. Mechanical properties were tested for native pig bladder, BAM, and BAM–collagen (1%)–cellulose (1%). Testing of the mechanical properties, such as tensile strength and elongation at break, of Young’s modulus was carried out using a Zwick Roell Z0.5 (Zwick Roell, Ulm, Germany). Appropriate sections were cut from the test samples. The static tensile test was carried out under the following assumptions: the clamping distance of the specimen between the jaws was 40 mm, and the gripping speed was 50 mm/min.

### Evaluation of Inflammatory Response to Graft *In Vivo*

Materials used for the creation of the composite were tested to determine the induction of inflammation: alone (BAM, collagen, cellulose) or with a combination (BAM + collagen, BAM + cellulose, BAM + collagen + cellulose). Materials were implanted subcutaneously in the pig model (*n* = 3). Under general anesthesia, small incisions on the pig side were made to form a subcutaneous pocket in which tested materials were placed for 1 month. After that time, materials were removed and submitted to histological examination (hematoxylin and eosin staining) to assess the inflammation.

### Permeability

To evaluate the permeability of the materials, fragments of BAM–collagen–cellulose composite (*n* = 4) or BAM (*n* = 4) were positioned atop a cylindrical tube, with the end containing the tested material sealed using a screw cap featuring an aperture. The assembly was then oriented with the material at the bottom, and the tube was filled with PBS. Permeability was assessed by measuring the volume of fluid that passed through the material after 1–6, 12, and 24 h.

### Cell Seeding Tests

To assess cell adhesion to composites (BAM–cellulose (1%), BAM–collagen (1%), BAM–collagen (1%)–cellulose (1%)), a cell seeding experiment was conducted. AD-MSCs suspension was divided into two aliquots for seeding: one was injected at regular intervals into the composite interior using an insulin syringe, while the second portion was applied to the surface of the composite fragments. The target seeding density was 10 × 10^6^ cells/cm^2^ of composite material. Cultures were incubated under standard conditions (37 °C, 5% CO_2_) for 7 days. Following this incubation period, the samples were fixed in a solution of 2.5% glutaraldehyde and 2% paraformaldehyde (pH = 7.4). Cell adhesion was analyzed using a scanning electron microscope (SEM).

### BAM–Collagen–Cellulose Composite Cytotoxicity

The cytotoxicity test of the material was conducted in an external accredited laboratory for biocompatibility testing of biomedical devices (Konmex Biolabs, Poland). The scope of the study included testing of the cytotoxic properties of the test item carried out following the principles of ISO 10993-5:2009­(E) and ISO 10993-12:2021­(E) standards. Triplicate monolayers of L929 cells were dosed with 600 μL of extracts (0.1 g/mL composite; 6 cm^2^/mL latex – positive control; 0.2 g/mL HDPE – negative control) and incubated in the presence of 5 ± 0.1% CO_2_. The staining solution was prepared directly before use by mixing the trypan blue solution with supplemented MEM in a 1:1 ratio. After incubation, 100 μL of prepared staining solution was dispensed in each well. The cytotoxic potential was assessed based on ISO 10993-5:2009­(E), Biological evaluation of medical devices – Part 5: Tests for in vitro cytotoxicity, Table 1 – “Qualitative morphological grading of cytotoxicity of extracts”.

**Table 1:** Elemental Analysis (Weight %)

Spectrum	C	N	O	Na	Mg	Al	Si	P	S	**Cl**	**K**	**Ca**	**Fe**	**Cd**	**Hg**	**Pb**
**BAM (weight %)**																
48494	40.63	2.86	41.09	0.44	0.75	1.07	0.84	0.06	0.35	0.43	0.37	6.25	0.58	0.08	1.39	2.19
48495	36.73	18.99	37.82	0.72	0.33	0.90	0.13	0.19	0.35	0.46	0.01	0.09	0.14	0.11	1.37	1.66
48496	37.21	18.82	37.99	0.60	0.35	0.77	0.12	0.13	0.35	0.35	0.00	0.07	0.14	0.10	1.25	1.74
48497	36.73	18.52	38.98	0.52	0.27	0.78	0.11	0.16	0.38	0.32	0.01	0.07	0.14	0.10	1.23	1.68
**BAM/oxidized cellulose (weight %)**																
48523	22.97	15.66	42.08	6.98	0.22	0.96	0,08	0.78	0.21	5.20	0.07	1.38	0.16	0.10	1.27	1.87
48524	23.79	16.71	40.75	7.07	0.42	1.21	0,15	0.82	0.29	3.68	0.06	1.34	0.19	0.13	1.53	1.86
48525	22.59	1528	40.40	8.20	0.40	1.28	0,15	0.84	0.24	4.64	0.08	1.48	0.15	0.14	1.77	2.37
48526	18.57	13.22	48.09	8.27	0.52	1.10	0,63	1.37	0.12	2.92	0.06	2.45	0.13	0.11	0.97	1.46
**BAM/collagen (weight %)**																
48509	17.89	16.06	45.47	9.03	0.22	0.81	0,15	0.36	0.13	5.55	0.05	0.03	0.14	0.05	1.00	1.58
48510	15.18	15.33	46.72	10.08	0.01	0.86	0,00	0.32	0.11	8.41	0.06	0.04	0.13	0.00	1.15	1.60
48511	21.35	16.85	41.57	8.81	0.28	1.36	0,17	0.39	0.23	5.44	0.04	0.06	0.20	0.08	1.31	1.86
48512	18.68	16.86	45.19	8.50	0.11	0.98	0,03	0.29	0.14	6.43	0.05	0.04	0.14	0.03	1.18	1.35
**BAM/collagen/cellulose (weight %)**																
48502	17.85	10.60	46.95	9.24	0.11	0.38	0,10	1.51	0.05	7.93	0.13	2.28	0.10	0.06	1.18	1.54
48503	20.27	12.56	45.60	10.28	0.36	0.74	0,13	0.72	0.08	5.91	0.10	0.62	0.11	0.10	0.96	1.46
48505	22.03	14.01	46.30	7.62	0.21	0.61	0,07	0.69	0.13	4.97	0.09	0.71	0.12	0.06	0.96	1.40

### Intracutaneous Reactivity Test

The intracutaneous reactivity test was conducted in an external accredited laboratory for biocompatibility testing of biomedical devices (Konmex Biolabs, Poland). The study aimed to assess the skin irritation potential of the test item, following the principles outlined in the ISO 10993-10:2021­(E) standard. The evaluation was performed using albino rabbits, with one group of three rabbits. The left side of each rabbit was treated with the test item extracts, while the right side was treated with the relative control solvents (Sodium Chloride Injection and Cottonseed Oil). The animals were observed immediately after injection and at 24 ± 2, 48 ± 2, and 72 ± 2 h post-treatment to assess local reaction signs. Injection sites were examined for tissue responses, such as erythema, edema, and eschar.

### Acute Systemic Toxicity

The acute systemic toxicity test was conducted in an external accredited laboratory for biocompatibility testing of biomedical devices (Konmex Biolabs, Poland). The study aimed to evaluate the toxicity potential of the test item following the principles of the ISO 10993-11:2017­(E) standard. The evaluation was performed using house mice (BALB/C, females), with four groups of five animals each. The animals in each group were injected with 50 mL/kg of the following extracts: polar (sodium chloride), nonpolar (cottonseed oil), and controls for both polar and nonpolar solvents. The extracts and control solutions were administered intraperitoneally. Clinical examinations and body weight measurements were conducted 24 ± 2, 48 ± 2, and 72 ± 2 h after injection.

### Mutagenic Potential Assay – AMES Method

The mutagenic potential assay was conducted in an external accredited laboratory for biocompatibility testing of biomedical devices (Konmex Biolabs, Poland). The scope of the study included testing of the mutagenic properties of the test item carried out in accordance with the principles of ISO 10993-3:2014­(E), ISO 10993-12:2021­(E), and ISO 10993-33:2015 standards, and OECD 471 guideline. Bacteria were exposed to full-strength extracts of the test item, as well as positive and negative controls, for 90 min in a medium containing sufficient histidine () or tryptophan (). After exposure, the cultures were diluted in a pH indicator medium lacking histidine or tryptophan. After 2 days, cells that have undergone reversion to amino acid prototrophy grow into colonies. Bacterial metabolism reduces the pH of the medium, changing the color of the well. The number of wells containing revertant colonies was counted for each group (test item and positive control) and compared to the solvent (negative) control. The mutagenic potential of samples was assessed directly and in the presence of 4.5% of liver Aroclor-induced S9 fraction. Baseline, fold increase over baseline value, and binominal B-value were calculated using an Excel spreadsheet provided by the manufacturer (Calculation Sheet for AMES MPF Penta 2, version 1.00, Xenometrix).

### Computational Modeling

A numerical analysis using the finite element method (FEM) in the LS-Dyna solver (ANSYS, USA) was prepared for preliminary assessment of the graft shape. The prepared numerical model allowed for assessing the influence of the graft shape on the behavior of the bladder wall during the filling process and the possibility of cell migration from native tissue to the graft center.

The first step was to develop a material model of the native bladder tissue. For this purpose, experimental tests were implemented, which were used to calibrate the constitutive model of the material. Strength tests implemented using an Instron 5966 testing machine (Instron, USA), were mapped in the FEM environment. A 70 × 15 mm bladder tissue specimen was meshed using the 4-node quadrilateral Belytschko–Tsay shell element with an average dimension of 0.5 mm. The MAT_181_Simplified_Rubber/Foam material constitutive model was adopted for the analysis.

After developing constants for the constitutive model of the material, the geometry of the bladder was prepared based on CT urinary bladder images to ensure a realistic anatomical representation. The process of developing the full bladder model (Figure 3A,c) involved taking CT images (Figure 3A,a), and their optimization (Figure 3A,b). The final step was discretization of the created model (divided into finite elements; Figure 3A,d,e). SpaceClaim (ANSYS, USA) and Meshmixer (Autodesk, USA) softwares were used to optimize and prepare the bladder surface model, while the discretization of the model was done using LS-PrePost preprocessor (LSTC, USA). The type and size of the finite elements and the material model were implemented from the specimen model using the MAT_181_Simplified_Rubber/Foam constitutive model, calibrated based on experimental tensile tests of the native bladder tissue.

**Figure 3:**
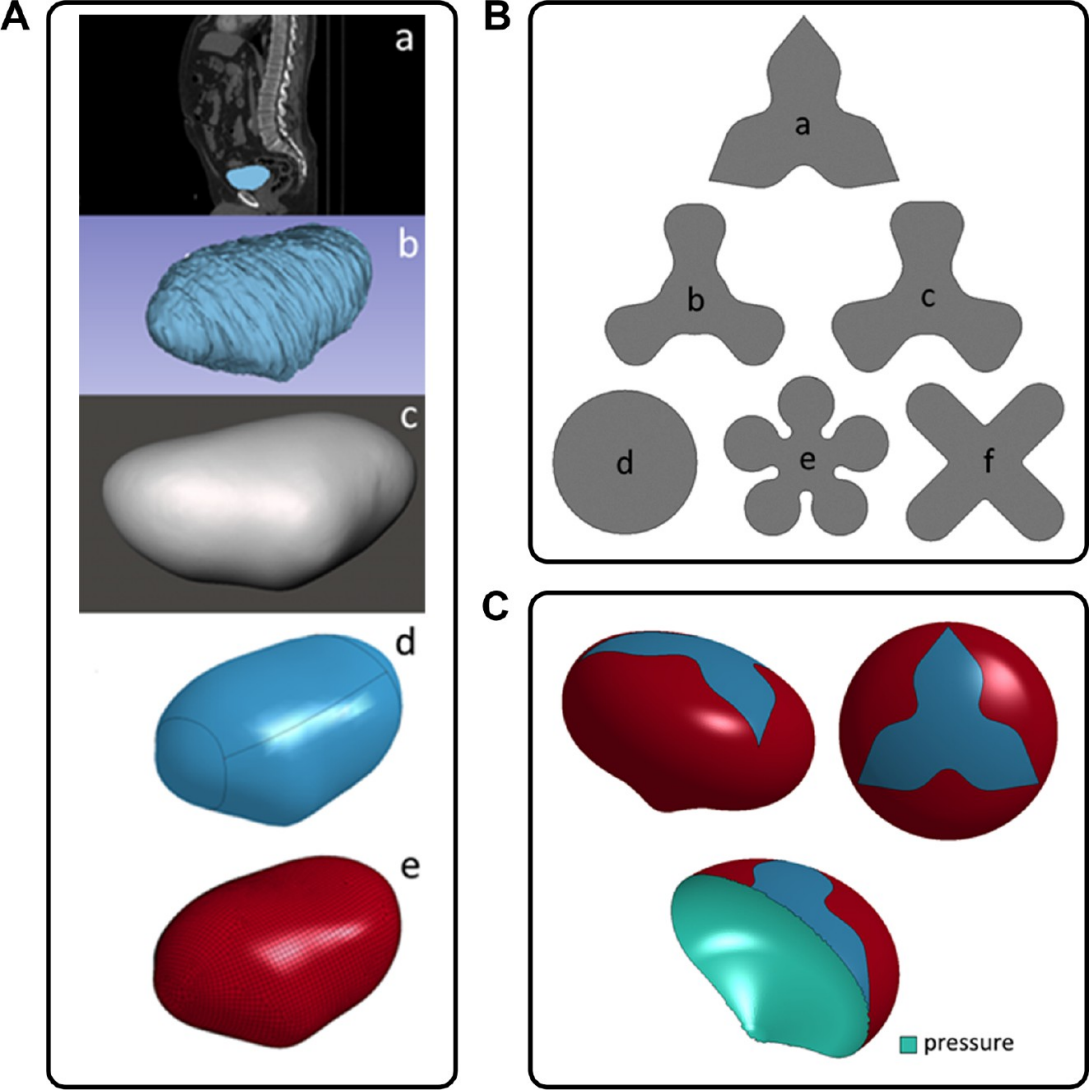
(A) Algorithm for proceeding to obtain a finite element mesh describing the geometry of the analyzed organ from CT images; (B) tested graft shapes; (C) bladder FEM model with the graft.

Numerical analysis was also performed to select the optimal shape of the graft on the basis of theoretical cell migration to its center. For this purpose, a numerical model of graft with different shapes was prepared (Figure 3B). The cell migration was simulated by the process of heating of models surface from the outer edges to the center under identical conditions for each shape. These analyses were also carried out using LS-Dyna software, assuming the model parameters as in the earlier analyses.

The bladder filling process was simulated by applying a pressure of 19.6 cm H_2_O in the normal direction to the mesh elements forming the bladder wall. The simulations assumed free expansion of the bladder wall under the applied pressure, with no additional mechanical constraints, except those resulting from the implant geometry and the calibrated material model. For numerical simulation of specimens used for validating material model, and discrete element size, boundary conditions were as follows: for the modeled specimens’ bottom edge, all degrees of freedom were taken away—both translational and rotational. A displacement in the longitudinal direction at a speed of 0.8 mm/s was applied to the upper edge. The bladder model with the graft is shown in Figure 3C.

For the concrescence analysis, heating simulations were performed to mimic the surface concrescence formation process. Identical heating conditions were applied for each tested implant shape to ensure comparability.

### Preclinical Study in a Porcine Model—A Graft Shape Selection for Augmentation Cystoplasty

Eight female pigs (weighing 30–35 kg) were used in the experiment. Animals were divided into 3 groups, in which three different prototypes were tested to assess their surgical implantability: 4-armed (*n* = 2), 3-armed (*n* = 3), and modified 3-armed (target shape; UROGRAFT; *n* = 3) (Figure 3B,a,c,f). Augmentation cystoplasty was performed under general anesthesia (ketamine 5 mg/kg, xylazine 1–3 mg/kg, butorphanol 0.1–0.2 mg/kg). For the procedure, the anesthetized animal was placed in a horizontal position. The surgical field was disinfected and draped with sterile drapes. The skin and fascia were incised in the median line of the lower abdomen, along a length of about 10 cm. After opening the peritoneal cavity, the urinary bladder was manually emerged. Bladder underwent incision (“+” or “Y” shape, depending on material type) on the bladder dome. After that, bladder augmentation with 30 cm^2^ of the tested composite was performed. The animals were sacrificed by intravenous administration of pentobarbital overdose (100 mg/kg) at the end of the 3-month follow-up. Macroscopic and photographic analyses of the graft on the filled bladder were performed. Harvested urinary bladders were placed in 10% buffered formalin solution and subjected to macroscopic, histological (HE and TM), and immunohistochemical (PAN-cytokeratin, SMA, CD31, S100) examinations. Urinary bladder regeneration was evaluated in two regions: graft center (C), and graft arm (A). The regenerated urinary bladder wall was compared with the native bladder wall structure (N). Morphometric analysis of urothelial layer thickness and the number of blood vessels (below 20 μm in diameter on 0.01 cm^2^ surface) was performed using EPview 1.3 software (Olympus, USA), and smooth muscle layer content was analyzed using ImageJ 1.52a software (NIH, USA).

### Culture of a Cell-Seeded BAM–Collagen–Cellulose Composite in a Bioreactor

To prepare tissue-engineered graft for augmentation cystoplasty, the BAM-collagen-cellulose (BAM-CC) composite, in its designated shape (UROGRAFT), was seeded with AD-MSCs. To achieve greater control and repeatability of the process, the ZRP Cultivation System (Zellwerk GmbH, Germany) was utilized in combination with a custom perfusion bioreactor. The seeding procedure began by placing the UROGRAFT into a specialized frame (Figure 4a,b), which is an integral component of the bioreactor. AD-MSCs suspension from the third passage was divided into two aliquots: the first portion was injected at regular intervals into the interior of the composite using an insulin syringe, while the second portion was applied to the surface of the composite fragments. The target seeding density was 10 × 10^6^ cells/cm^2^ of composite material. After seeding, the frame containing the composite was placed in an incubator for 1 h to allow cell attachment to the surface. Subsequently, the frame was transferred to the bioreactor vessel (Figure 4c,d). Once connected to the ZRP platform, the bioreactor vessel was filled with culture medium (Dulbecco’s Modified Eagle Medium/Ham’s F12 (DMEM/Ham’s F12, Corning, USA) supplemented with 10% fetal bovine serum (FBS, Biowest, USA), 10 ng/mL basic fibroblast growth factor (b-FGF, Gibco, USA), 100 U/mL penicillin, 100 μg/mL streptomycin (HyClone, USA), and 5 μg/mL amphotericin B (Corning, USA)). The frame with the composite was set in rotation (0.8 rpm) inside the culture vessel, and the culture was maintained under the continuous circulation of the culture medium (5 mL/min). Fresh culture medium was continuously supplied to the vessel, with the flow rate regulated by the bioreactor system based on glucose concentration in the culture medium. Throughout the culture process, temperature, pH, and oxygen concentration were continuously monitored.

**Figure 4:**
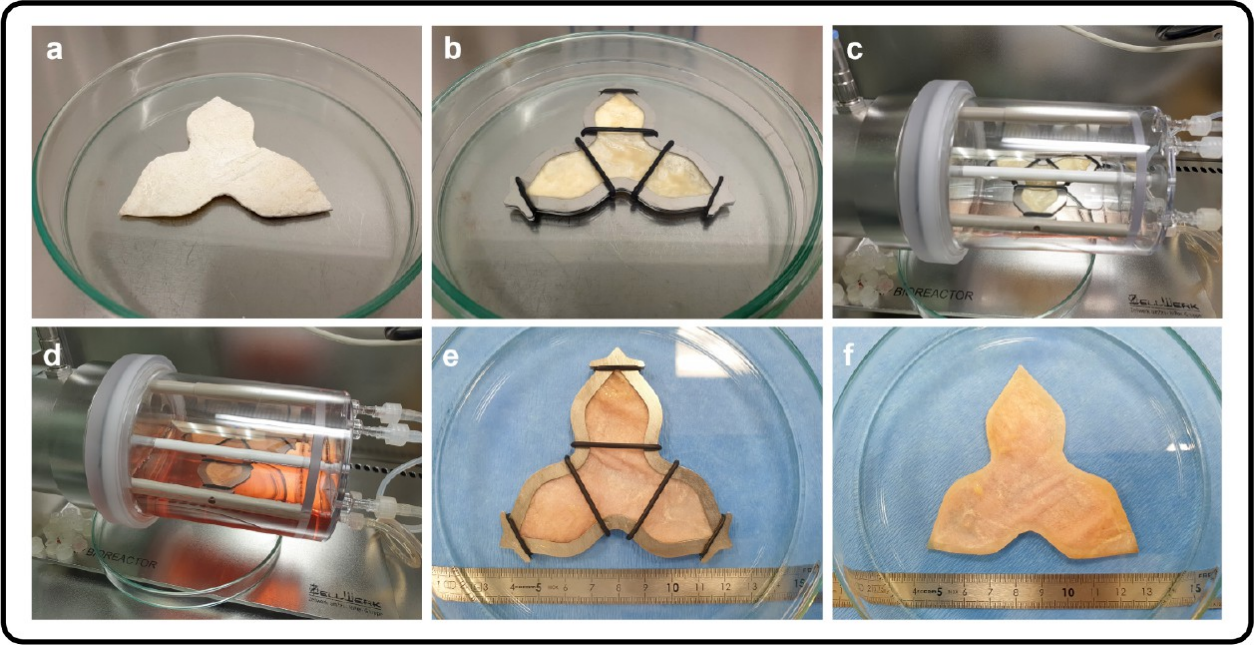
Preparation of tissue engineered graft for augmentation cystoplasty (a) manufactured BAM-CC composite in its final shape (UROGRAFT), (b) UROGRAFT placed in the bioreactor frame and seeded with AD-MSCs, (c,d) frame with seeded UROGRAFT placed in the perfusion bioreactor vessel connected to the Zellwerk’s ZRP platform, (e,f) UROGRAFT seeded with AD-MSCs following 7-days of a dynamic culture.

After 7 days of culture (Figure 4e,f), the UROGRAFT seeded with AD-MSCs samples were fixed in a solution containing 2.5% glutaraldehyde and 2% paraformaldehyde (pH 7.4) and analyzed using a scanning electron microscope (JEOL JSM-6390LV, Japan). Additionally, some fragments were fixed in a 4% formaldehyde solution and stained with the LIVE/DEAD cell viability kit (Thermo Fisher Scientific, USA). The stained samples were then analyzed using a confocal microscope (LSM 900, Zeiss, Germany).

### Statistical Analysis

All data were presented as means ± SD. The normal distribution of data was analyzed using the Shapiro–Wilk test, and in circumstances where a normal distribution was not observed, the Kruskal–Wallis test was employed. Parametric analysis was performed with one-way ANOVA with Tukey post hoc or 2-way ANOVA (GraphPad Software 8.4., USA).

## Results

### Effectiveness of Decellularization

HE analysis showed that the tissue structure was well preserved in the native samples, whereas the BAM fragments manufactured by protocols I, II, and III showed some degree of disorganization (Figure 5A). All BAMs extruded by protocols I–III showed a decrease in the number of nuclei and DNA compared to native bladder tissue. However, there were important differences when comparing the three protocols. Analysis of the number of nuclei in HE samples showed that the least effective protocol in terms of cell removal was that based on 24-h incubation with 1.0% Triton X-100 (protocol I), with a mean number of nuclei of 86.2 ± 25.55, compared to 38.98 ± 12.61 for the method using 1% SDS (protocol II) and no nuclei in the image for the protocol using a combination of Triton X-100 and SDS (protocol III). It was also observed that the lowest number of nuclei were observed in the samples from RI – 59.8 ± 14.60 (protocol I), 24.73 ± 24.8 (protocol II), and the highest from RIII – 110 ± 19.64 (protocol I), 48.73 ± 36.42 (protocol II). For protocol III, no nuclei were detected for all RI–RIII regions (Figure 5A). Based on these analyses, protocol I was excluded from further analysis.

**Figure 5:**
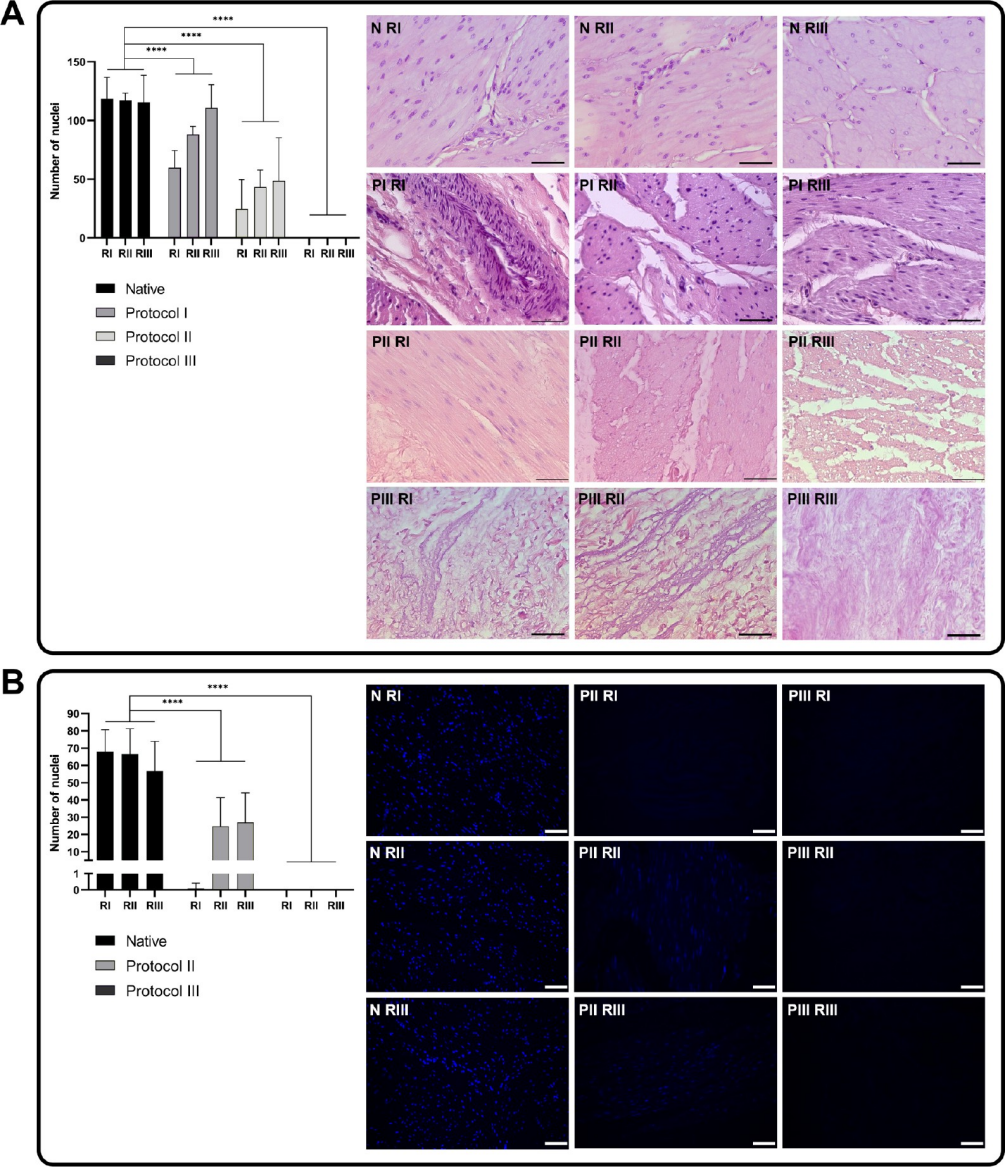
(A) Decellularization efficiency: comparison of the number of nuclei in samples, representative images of hematoxylin and eosin staining of individual regions (RI–III) of native porcine urinary bladder (*N*) and bladders decellularized with protocols I–III (PI–III) (40×, bar = 50 μm) ****p < 0.001; (B) comparison of the number of nuclei in samples, representative images of DAPI staining for individual regions (RI–III) of native porcine bladder (*N*) and bladders decellularized with protocols I–III (PI–III) (40×, bar = 75 μm) ****p < 0.001.

A similar observation was made with 4′,6-diamidino-2-phenylindole (DAPI) staining—a significant reduction in the number of nuclei in BAM manufactured by protocol II (17.31 ± 14.97) and protocol III, with protocol III showing the most extreme reduction—no nuclei were detected for any of the regions (RI–III), indicating that the cells were effectively removed during the decellularization process. The decellularization process was most effective at the top of the bladder (RI for protocol II was 0.083 ± 0.334) (Figure 5B). Protocol III was, therefore, considered the most effective as it completely removed the cells, ensuring the acellularity of the extracellular matrix and its potential for further use in tissue regeneration. Although still effective, protocol II showed some cell loss but retained some cellular structure.

DNA quantification was performed to assess the effectiveness of decellularization protocols in removing cellular DNA from tissue samples. The results showed significant differences in DNA content between native tissue and decellularized samples. A significant decrease in DNA content was observed in bladder tissue after decellularization. Among the protocols tested, protocol III was found to be the most effective in terms of DNA removal, with a DNA content of only 9.72 ± 2.89 ng DNA/mg dry weight. This result represents a significant reduction in DNA content and highlights the protocol’s effectiveness in removing cellular components. In comparison, the results showed that protocol II had a higher DNA content of 35.07 ± 2.358 ng DNA/mg, indicating a less advanced state of decellularization. However, this level was still lower than in the native tissue. Notably, the native tissue showed an exceptionally high DNA concentration of 3813 ± 554.9 ng DNA/mg dry weight, highlighting the significant DNA reduction achieved by both decellularization protocols (Figure 6A). The results showed a significant statistical difference (*p* < 0.01) between native tissue and protocol III, which suggests that protocol III is the most effective in removing cellular DNA, providing a biomaterial with minimal residual genetic material, which is crucial for tissue engineering and regenerative medicine applications.

**Figure 6:**
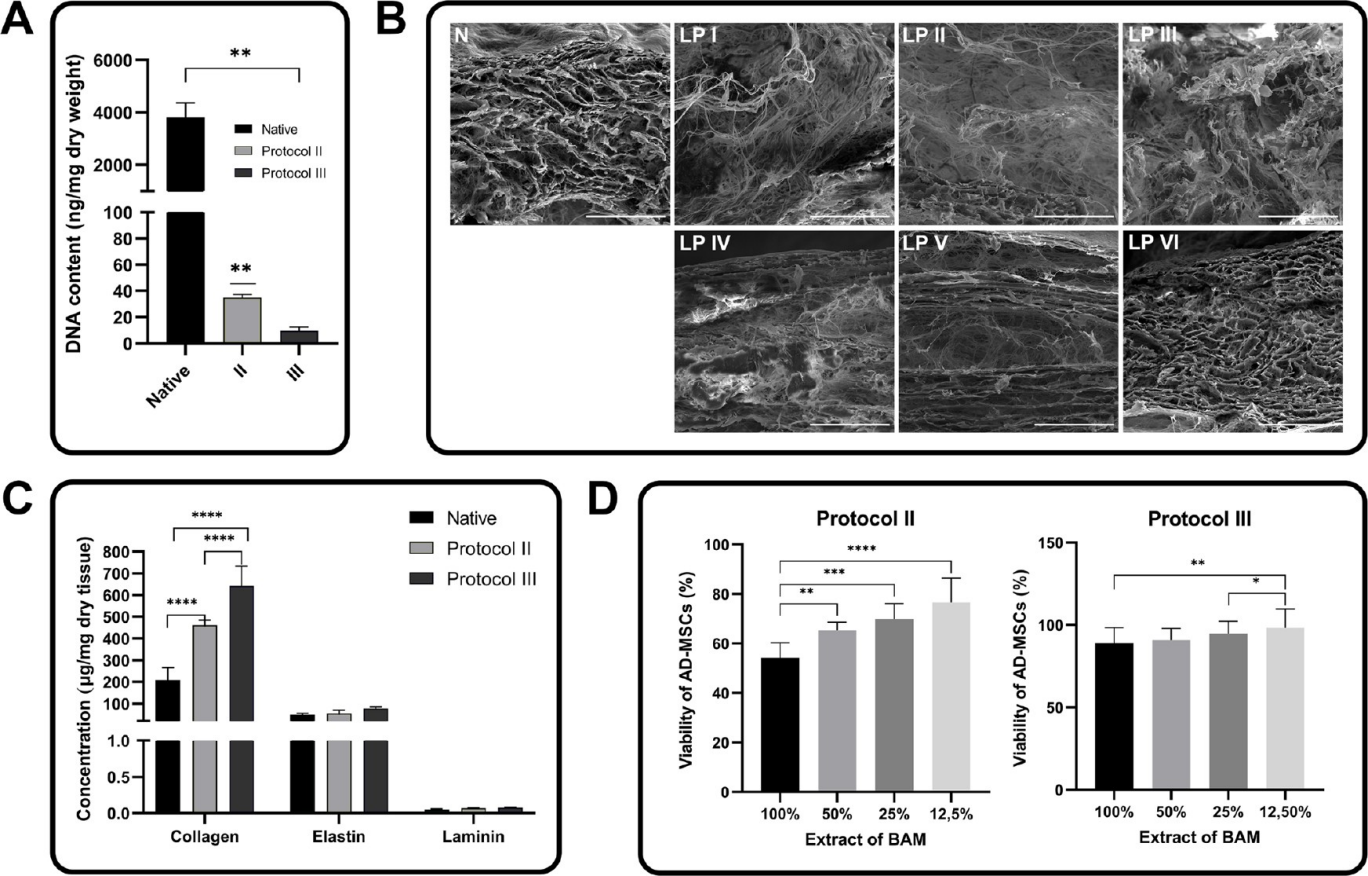
(A) Comparison of DNA content in native bladder (N) and bladder decellularized with protocol II–III (PII–III) [ng/mg dry weight]; (B) SEM analysis results of lyophilization protocols (LP I–VI) of BAM produced by protocol III and native bladder (N) bar = 200 μm; (C) comparison of the concentration of extracellular matrix (ECM) proteins—collagen, elastin and laminin in native bladder (N) and bladder decellularized with protocol II–III (PII–III) [μg/mg dry weight]; (D) comparison of AD-MSCs viability results for BAM extracts (100%, 50%, 25% and 12.5%) produced by protocol II and III after 24 h; **p < 0.1, ****p < 0.001.

### Lyophilization

Freeze-drying at 0.120 mbar and −40 °C for 17 h, preceded by freezing the material at −20 °C for 2 h (LP V), was shown to be the most effective method (Figure 6B). This protocol allows for the successful preparation of a homogeneous 3D structure with comparable pore sizes, not exceeding 300 μm. In contrast, samples prepared by the other methods resulted in heterogeneous structures, with large variations in pore size in different locations throughout the BAMs.

Analysis in the SEM confirmed the cell-free nature of the BAM scaffolds produced by protocol III. The resulting BAMs were shown to have high porosity. The structure of the collagen fibers was preserved. The outer layer of collagen fibers was not affected—the fibers were smooth, indicating that the decellularization process does not damage collagen fibers (Figure 6B).

### Quantification of ECM Component

The analysis of extracellular matrix (ECM) proteins—collagen, elastin, and laminin—content in lyophilized BAMs revealed that protocol III showed the highest protein concentration per mg of tissue dry weight. Specifically, protocol III expressed 642.3 ± 90.57 μg/mg of collagen, 77.62 ± 7.43 μg/mg of elastin, and 77.88 ± 3.87 ng/mg of laminin. This was significantly higher compared to the values from lyophilized native bladder tissue, which contained 207.99 ± 57.29 μg/mg of collagen, 48.30 ± 7.49 μg/mg of elastin, and 50.85 ± 7.09 ng/mg of laminin. Protocol II, in comparison, showed intermediate values, with 461.73 ± 22.23 μg/mg of collagen, 54.72 ± 14.83 μg/mg of elastin, and 67.18 ± 8.28 ng/mg of laminin (Figure 6C).

Statistical analysis of these data showed a significant difference (*p* < 0.001) between native bladder tissue and protocol III for collagen, indicating more efficient retention of this ECM component in the decellularized scaffolds produced by protocol III (Figure 6C). These results suggest that protocol III not only effectively decellularized bladder tissue, but also maintains a higher content of a key ECM protein, making it the most suitable protocol for producing BAM for tissue engineering applications.

The cytotoxic effects of extracts from porcine BAMs manufactured according to protocols II and III on AD-MSCs were assessed by MTT. Extracts from lyophilized BAMs were prepared at four concentrations: 100%, 50%, 25%, and 12.5%. The results obtained after 24 h of exposure showed high levels of cell viability at all concentrations tested for protocol III and reduced viability for protocol II (Figure 6D). The average cell viability for the 100% BAM extract manufactured according to protocol III was above 85% (88.90 ± 9.42%), thus confirming that matrices obtained using this protocol can be considered as noncytotoxic. In contrast, the average cell viability for 100% BAM extracts manufactured according to protocol II was 54.32 ± 5.99%. This value is below the 70% viability threshold that is stated by ISO 10993-5, below which the material displays cytotoxic potential.

### Choosing the BAM Cross-Linking Method

SEM analysis showed that the BAM cross-linking with collagen (2%) or cellulose (5%, 10%) has not reduced pore size (Figure 7A). Therefore, the composites BAM–collagen (1%), BAM–cellulose (1%) and BAM–collagen (1%)–cellulose (1%) were selected for further analysis.

**Figure 7:**
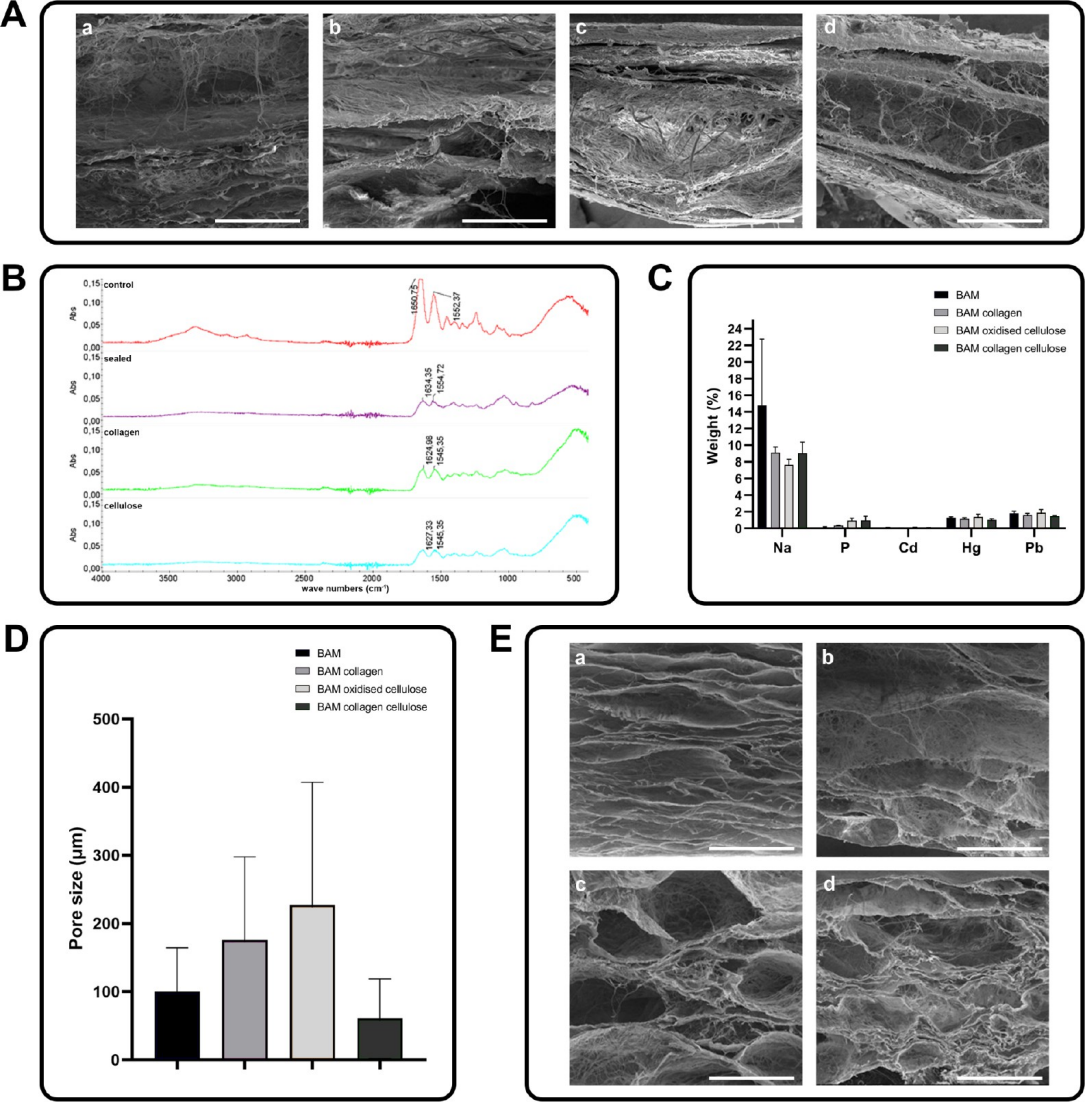
(A) SEM analysis results: a—BAM, b—BAM–collagen (2%), c—BAM–cellulose (5%), d—BAM–cellulose (10%); (B) ATR-FTIR spectrum for BAM (red line) and BAM–collagen (1%) (green line), BAM-oxidized cellulose (1%) (blue line), BAM–collagen (1%)–oxidized cellulose (1%) (purple line); (C) elemental analysis (weight %) for BAM (blue), for BAM–cellulose oxidized (1%) (orange), for BAM–collagen (1%) (pink) and BAM–collagen (1%)–cellulose (1%) (green); (D) average pore size (μm): for BAM (blue), for BAM–cellulose oxidized (1%) (orange), for BAM–collagen (1%) (pink) and for BAM–collagen (1%)–cellulose (1%) (green); (E) Scanning electron microscope images of decellularized walls of pig urinary bladders: a—BAM, and for composites: b—BAM–cellulose oxidized (1%), c—BAM–collagen (1%), d—BAM–collagen (1%)–cellulose (1%).

Analysis using attenuated total reflectance Fourier-transform infrared spectroscopy (ATR-FTIR) showed that cross-linking occurs between BAM and the polymers, i.e., collagen and cellulose, used separately as well as together. The highest value of area under the peak (poor cross-linking) at 1580–1710 cm^–1^ was obtained for BAM, −6.950, and the lowest value (good cross-linking) for the BAM–collagen–cellulose composite, −0.968 (Figure 7B). This means that the BAM–collagen (1%)–cellulose (1%) composite is better cross-linked than the BAM–collagen (1%) or BAM–cellulose (1%) composites. Furthermore, the BAM–collagen (1%)–cellulose (1%) composites had a smaller pore size than BAM, BAM–collagen (1%) and BAM–cellulose (1%) (Figure 7D–E).

The BAM–collagen (1%)–cellulose (1%) composite had a lower content of heavy metals such as Cd, Hg, and Pb than BAM alone or the other composites tested (Figure 7C and Table 1). The increase in elements such as Na, Cl, and P is related to using the PBS solution during the cross-linking steps.

Based on the study, the BAM–collagen (1%)–cellulose (1%) composite (BAM–CC) was selected as the material with the most promising properties. In order to understand its potential in more detail, it was decided to juxtapose it with BAM in subsequent comparative tests. This analysis will allow an assessment of the extent to which additives such as collagen and cellulose influence the material’s properties. Based on differential scanning calorimetry (DSC), it can be seen that in the first stage of the study, both BAM and BAM–collagen (1%)–cellulose (1%) give up freely bound water (up to 120 °C), which was taken up from the environment after the freeze-drying process. Then, for the BAM–collagen (1%)–cellulose (1%) composite, at peaks (122 °C; 134.5 °C), degradation of the un-cross-linked parts of the collagen and oxidized cellulose takes place, the actual degradation starting at 210 °C. For BAM, proper degradation starts at 223 °C (Figure 8B). Although the BAM–collagen (1%)–cellulose (1%) composite shows a lower degradation onset temperature (210 °C) compared to pure BAM (223 °C), this difference does not significantly affect its potential clinical application. The temperatures reached under clinical conditions and in the human body are significantly lower than the degradation temperatures of the material, meaning that the mechanical and functional properties of the composite remain entirely sufficient for its anticipated applications.

**Figure 8:**
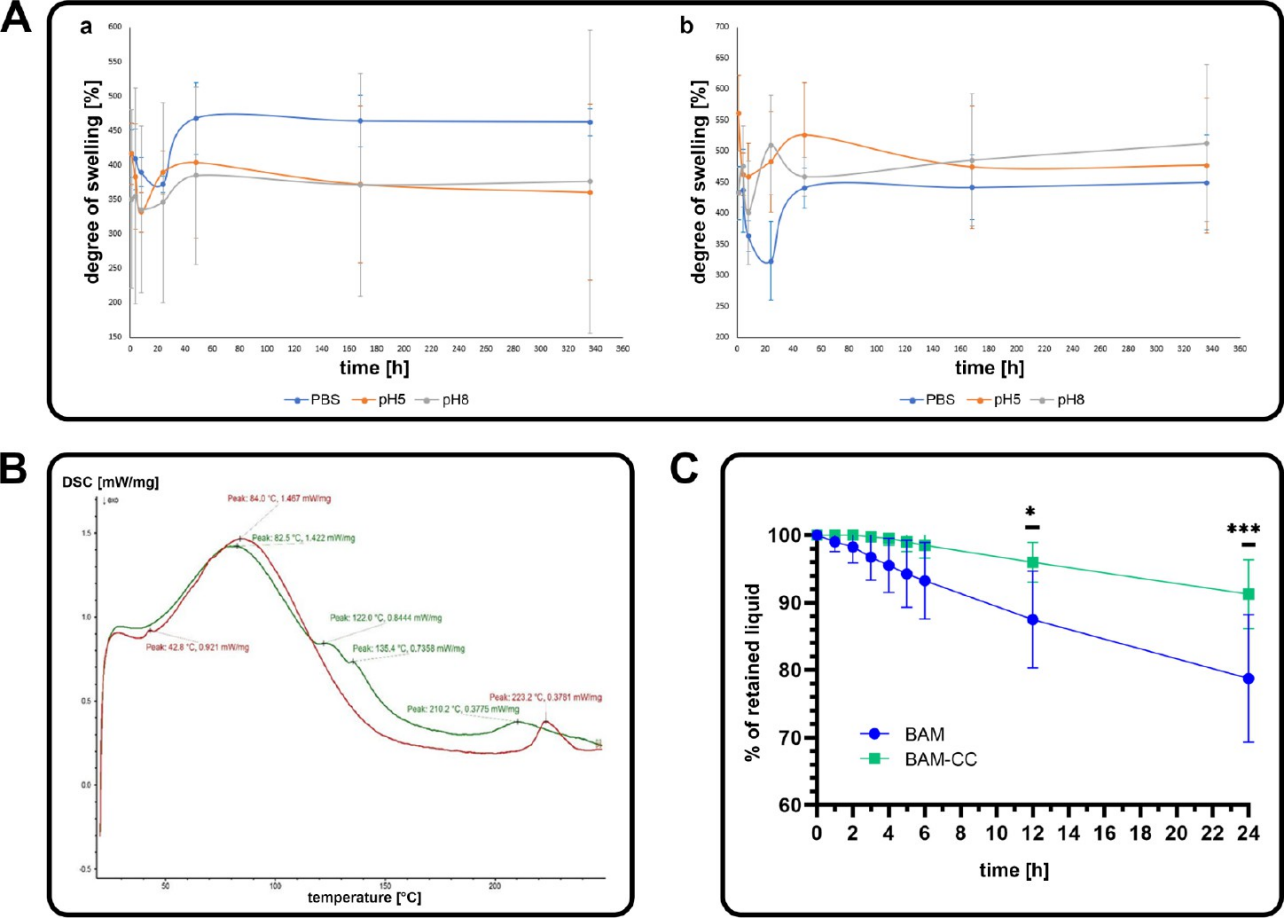
(A) Swelling graph for: a—BAM, b—BAM–CC composite in solutions: PBS (blue line), pH5 (orange line), pH 8 (green line); (B) DSC plot for: BAM (green line) and BAM–CC composite (red line); (C) permeability of BAM and BAM–CC composite over time (*p < 0.0296, ***p < 0.0003).

Based on swelling and degradation analysis, it can be concluded that both BAM and the BAM–collagen (1%)–cellulose (1%) composite are hygroscopic, increase their mass by at least four times their dry weight, and do not degrade within 2 weeks (Figure 8A).

Mechanical analysis showed that the BAM–collagen (1%)–cellulose (1%) composite was less elastic than native bladder and BAM but could still be characterized as an elastic material due to its low Young’s modulus value (Table 2).

**Table 2:** Mechanical Analysis Results for Native Pig Bladder, BAM, and BAM–Collagen (1%)–Cellulose (1%) Composite

		*E*_mod_ [GPa]	*F*_max_ [MPa]	d*L* at F_max_ [mm]	*F*_Bruch_ [N]	d*L* at destruction [%]	*a*_0_ [mm]	*b*_0_ [mm]	*S*_0_ [mm^2^]
Native pig bladder	*x* **®**	0.00000879	0.452	122.1	6.04	320,8	4.82	5.183	26.21
	*s*	0.00000396	0.0808	13.7	0.940	32,6	0.2523	0.2317	0.01
	*v*	45.03	17.88	11.22	15.57	10,15	5.23	4.47	0.05
BAM	*x* **®**	0.000496	5.80	63.1	2.84	231,3	0.159	5.866	0.96
	*s*	0.000156	1.58	10.3	0.698	47,2	0.01192	0.4355	0.13
	*v*	31.41	27.17	16.38	24.61	20,42	7.49	7.42	13.75
BAM–collagen (1%)–cellulose (1%)	*x* **®**	0.00307	26.8	18.0	3.78	65,8	0.04571	6.3	0.29
	*s*	0.000416	4.66	2.6	0.417	8,1	0.002215	0.8799	0.04
	*v*	13.53	17.40	14.44	11.02	12,38	4.84	13.97	15.28

### Permeability Testing

The permeability of the BAM and the BAM–collagen–cellulose composite was evaluated over a 24-h period by measuring the percentage of liquid that passed through each material at various time points (Figure 8C). BAM exhibited a marked decline in permeability over time (78.75 ± 9.46% after 24 h). In contrast, the BAM–CC composite maintained relatively stable permeability throughout the experiment. The reduction in the percentage of liquid passed was gradual and limited, with no significant drop over the 24-h period (91.25 ± 5.12%). Statistical analysis revealed that the composite membrane exhibited significantly higher permeability than the BAM membrane at 12 h (*p < 0.0296) and 24 h (***p < 0.0003).

### Inflammatory Response

Cellulose, collagen, and BAM cross-linked with both polymers—collagen and cellulose induced a slight granulomatous reaction. Strong granulomatous reactions with granulocytes and lymphocytes were observed in the case of BAM alone and BAM with collagen or cellulose (Figure 9A).

**Figure 9:**
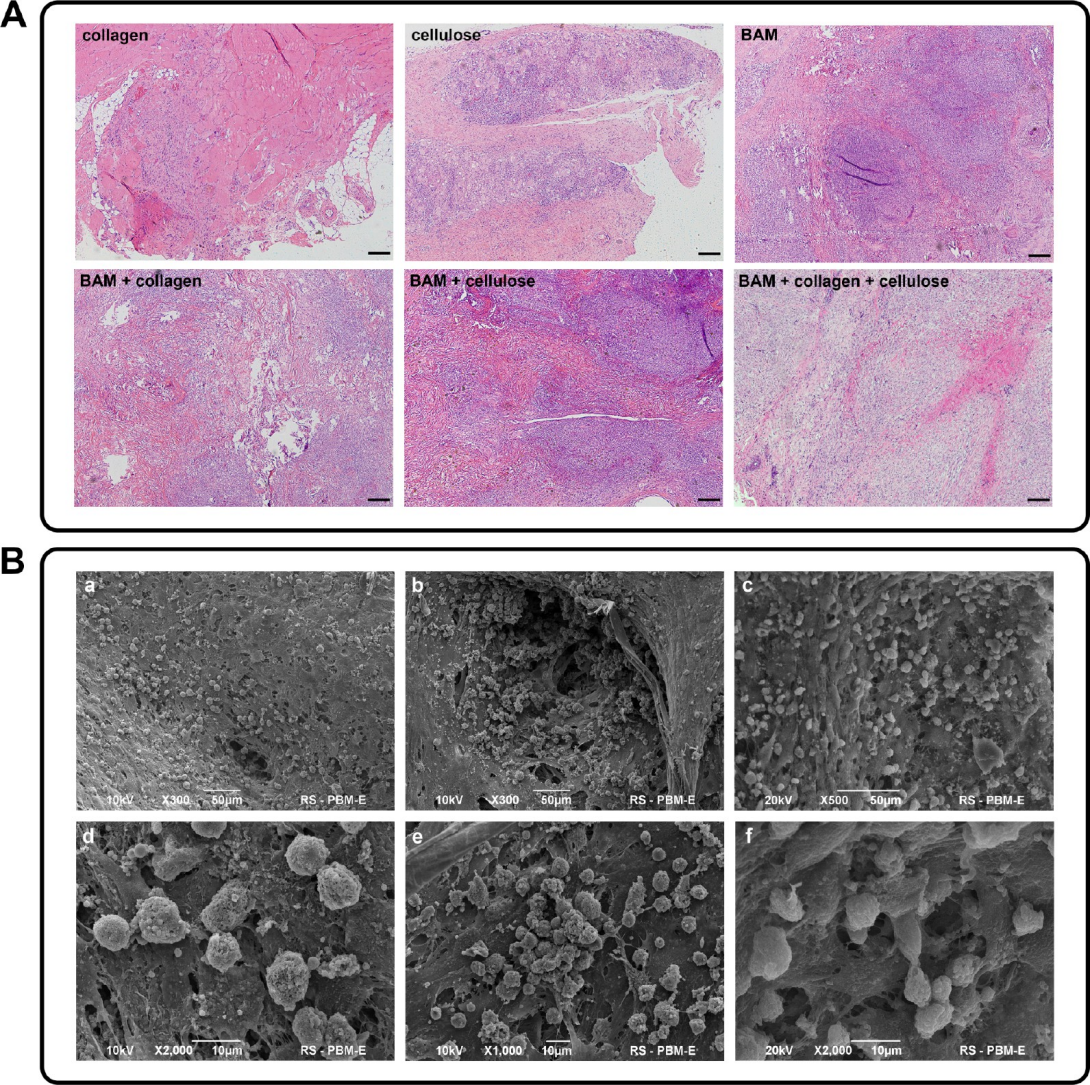
(A) Evaluation of inflammatory response—polymers used for bladder acellular matrix (BAM) cross-linking—collagen and cellulose were tested alone or in combination with BAM; light inverted microscope, scale bar = 100 μm; (B) scanning electron microscope images of composite fragments seeded with AD-MSCs: a, d—BAM–cellulose, b, e—BAM–collagen, c, f—BAM–collagen–cellulose.

### Possibility of Colonizing Composites with Cells

Scanning electron microscopy (SEM) analysis of composites: BAM–cellulose (1%), BAM–collagen (1%), and BAM–collagen (1%)-cellulose (1%) seeded with cells revealed that all materials supported cell attachment to their surface (Figure 9B). However, notable differences were observed in the cell density and the quality of the cells remaining on the surface of the composites. After 7 days of culture, the BAM–cellulose (1%) composite exhibited the lowest cell retention, with the cells displaying an irregular, porous structure of the cell membrane (Figure 9B,a,d). In contrast, the BAM–collagen (1%) and BAM–collagen (1%)–cellulose (1%) composites supported a higher number of cell habitats (Figure 9B,b,e,c, f). Notably, only the BAM–CC composite exhibited intact cell membranes, without evidence of porosity, suggesting a lack of cellular damage and cell adhesion visible as the growth of filopodia, cytoplasmic webbing, and flattening of the central mass (Figure 9B,c,f).

### Biocompatibility of BAM–Collagen–Cellulose Composite

Biocompatibility testing performed in accredited laboratory according to ISO standards showed that the BAM–collagen–cellulose composite is noncytotoxic (ISO 10993-5:2009), does not cause tissue reactions at injection sites (ISO 10993-10:2021), does not exhibit acute systemic toxicity in intraperitoneal and intravenous administration (ISO 10993-11:2017), and is not mutagenic (ISO 10993-3:2014). Biocompatibility testing ensures that biomaterials used for UROGRAFT manufacture are compatible with human tissues and do not cause unwanted side effects when in contact with them.

### Computational Modeling

The material model used in the FEM analysis (MAT_181_Simplified_Rubber/Foam) was calibrated and validated using experimental tensile tests performed on bladder tissue specimens. Two types of specimens were tested: one cut transversely and one cut longitudinally relative to the tissue structure. The comparison of experimental and numerical stress–strain results demonstrated a high level of agreement over the entire range of specimen elongation, as depicted in Figure 10A)

**Figure 10:**
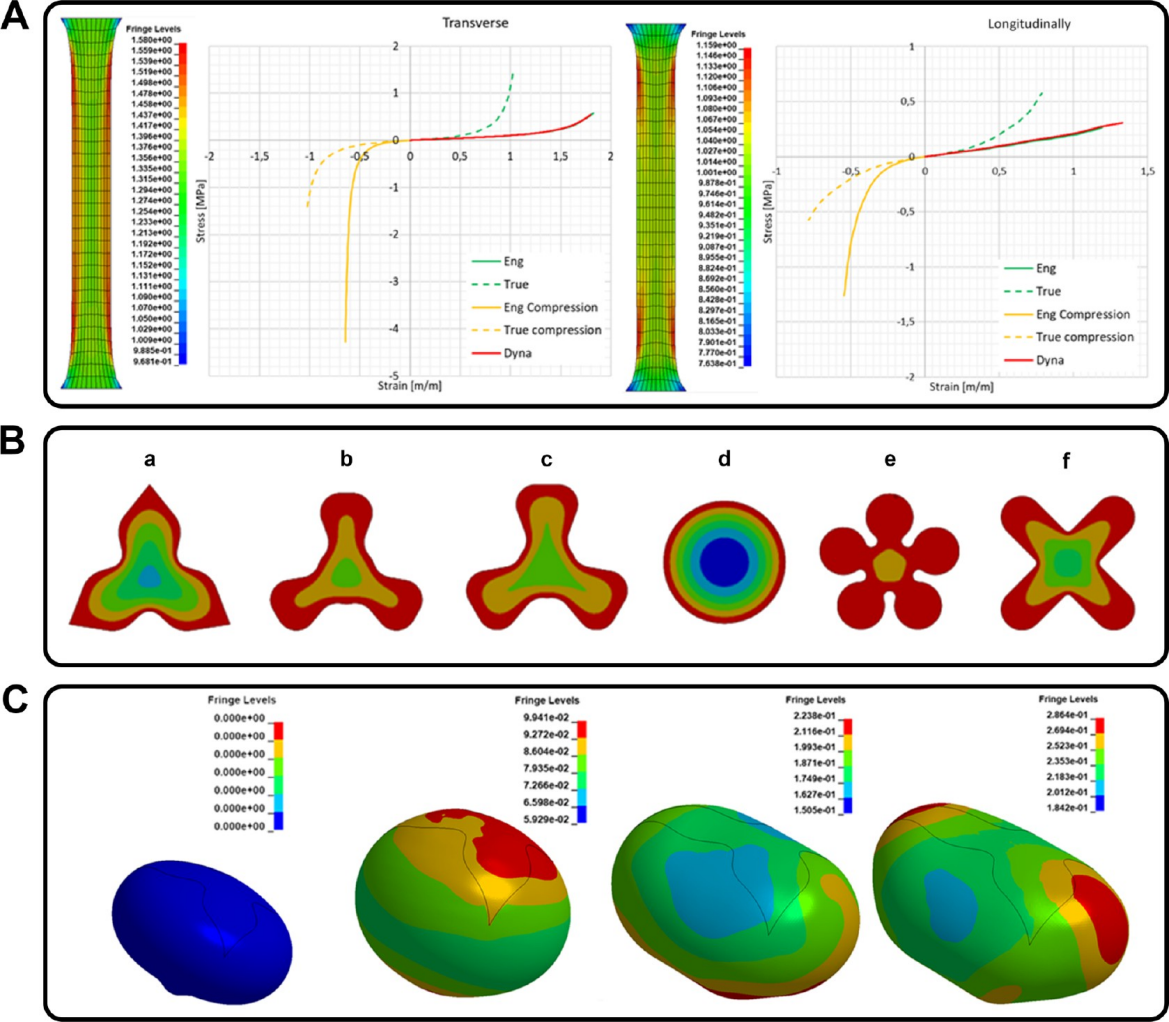
(A) Comparison of the experimental and numerical test; (B) concrescence occurrence on the implant; (C) stress distribution.

As a result of the simulation, it was shown that the most favorable heat distribution profile occurred on the implant shape shown in Figure 10B,e. While the least favorable, which was also confirmed by experimental studies, on the implant with the shape shown in Figure 10B,d. After consultation with surgeons, the shape presented in Figure 10B,e was rejected due to the impossibility of its implantation. That is why, in the first step, we decided to test the 4-armed shape presented in Figure 10B,f and the 3-armed shape presented in Figure 10B,c. Both shapes were eliminated after *in vivo* experiments due to difficulty in maintaining the shape of the graft after implantation. After computational modeling and *in vivo* results, the solution shown in Figure 10B,a was adopted for further consideration.

The final step of the numerical study was to analyze the effect of the shape of the implant on the distribution of Huber–Mises–Hencky reduced stresses. No negative impact of the implant was noted (Figure 10C).

### Selection of the Optimal Graft Shape for Augmentation Cystoplasty—A Preclinical Study

BAM-CC composite integrated well with the recipient tissues and microscopically was almost indistinguishable from the native bladder wall at the end of follow-up.

The implanted 4-armed BAM–CC composite graft after the end of the surgical procedure and after bladder filling changed the initial shape to a rectangle (Figure 11A). Therefore, in the second animal in this group, we used a graft with a larger (5 mm) margin, which made it possible to obtain a graft shape similar to the intended after augmentation. The additional margin increased the implant surface from 30 cm^2^ to 42 cm^2^. The graft obtained in this way has a surface that was too large in relation to the surface of the reconstructed bladder. The animal died 2 weeks after the surgery due to urinary bladder perforation and urine leakage.

**Figure 11:**
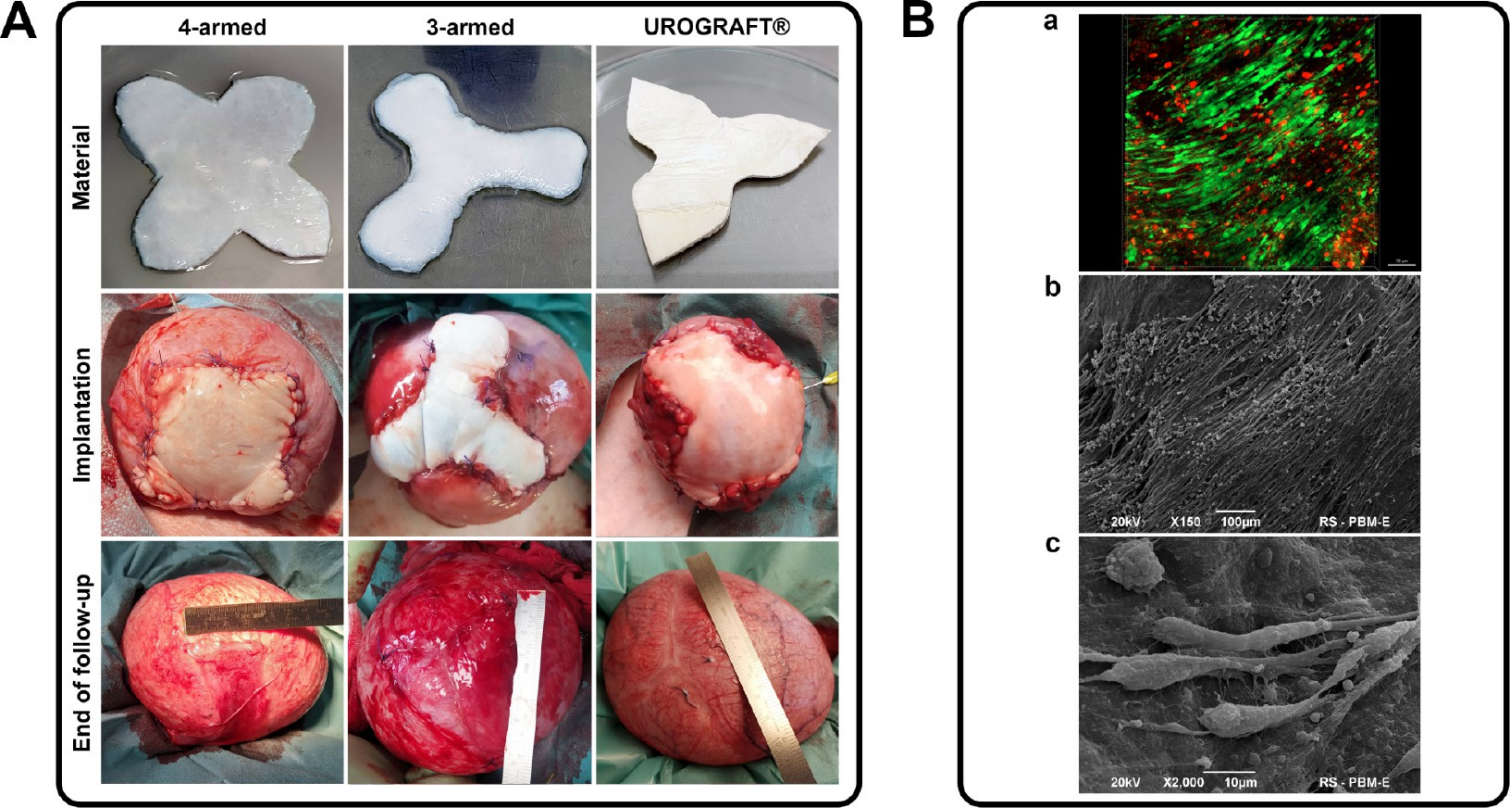
(A) Evaluation of scaffolds’ implantability—three different shapes, chosen in the previous part of the study, were tested. 4-Armed graft changed its shape to a rectangle, 3-armed in two cases to a circle, and UROGRAFT remained its initial shape after the end of follow-up in all cases; (B) adhesion ability of AD-MSCs to the UROGRAFT: cell viability tests using the LIVE/DEAD kit (green—live cells, red—dead cells), image of cells on the surface of the UROGRAFT after 7 days of culture in the bioreactor.

In the case of the 3-armed BAM–CC composite graft, the shape was maintained after implantation, however after the follow-up, the graft took on a spherical shape (Figure 11A). After filling the bladder, a “bulge” appeared, visible macroscopically in two cases, in one case adhesion with intestine was observed. The implanted material was separated from the host tissue by a clear scar in one case; in the second, the scar was not visible; in the third, the scar was visible only on the inner side of the bladder and had a Y shape corresponding to the graft shape.

The implanted graft in the shape of UROGRAFT maintains shape after augmentation and after the end of follow-up in all cases (Figure 11A). Reconstructed bladders exhibited normal typical urinary bladder oval shapes. No diverticula were observed. No adhesions with the intestine were noticed. Only a small scar, especially on the internal side of the implant, was visible, it had the shape of the letter Y, corresponding to the shape of the implanted material.

### UROGRAFT Cell Seeding and Bioreactor Culture Efficiency

AD-MSCs cultured on the UROGRAFT in a perfusion GMP bioreactor using the ZRP cultivation system were examined after 7 days via SEM and confocal microscopy (viability test). The cells remained firmly attached to the matrix (Figure 11B) and showed enhanced density of colonization compared to static cultures maintained in a standard incubator. An even distribution of cells was observed across the composite surface, forming a compact layer (Figure 11B,b). The cells exhibited a characteristic spindle morphology (Figure 11B,c), and no disruptions in the integrity of the cell membrane were detected. Furthermore, cell viability was maintained at a high level throughout the 7-day culture period (Figure 11B,a).

### Regeneration of Urinary Bladder Tissues

Histological and immunohistochemical analyses showed multilayered urothelium regeneration on the entire bladder wall augmented with BAM–CC composite. In the 3-armed BAM–CC group, the tissue layer was thicker; however, statistical differences were observed only between the arms of the 3-armed BAM–CC graft and arms of other tested groups and control (Figure 12B). Regeneration of the muscle layer was visible by Trichrome-Masson staining and smooth muscle actin (SMA) expression (Figure 12A). In the central part, there were places where the regeneration process was less advanced compared to the arms; the muscle fibers were smaller and less organized, and extensive connective tissue was visible between the fibers. Differences between tested groups were not visible; however, in the 4-armed BAM–CC group and center of the 3-armed BAM–CC group, muscle fibers were the least organized, while in the other tested groups (arm of 3-armed BAM–CC group and arm and center of UROGRAFT group), muscle fibers looked more like in native tissue (Figure 12A,B). Loosely arranged collagen fibers probably constitute a remnant of the implanted matrix (Figure 12A). The CD31 marker showed the presence of blood vessels in the bladder stroma both in the central and arm area of grafts. The highest number of newly created blood vessels (between 20 μm in diameter) were observed in the arm area of the UROGRAFT (Figure 12A,B). S100 staining confirmed nerve regeneration, mainly in the arm area of tested grafts. A lack of S100-positive cells was observed in the 3-armed BAM–CC group. In other tested groups, 1 to 5 nerves were observed in the field of view (Figure 12A, B).

**Figure 12:**
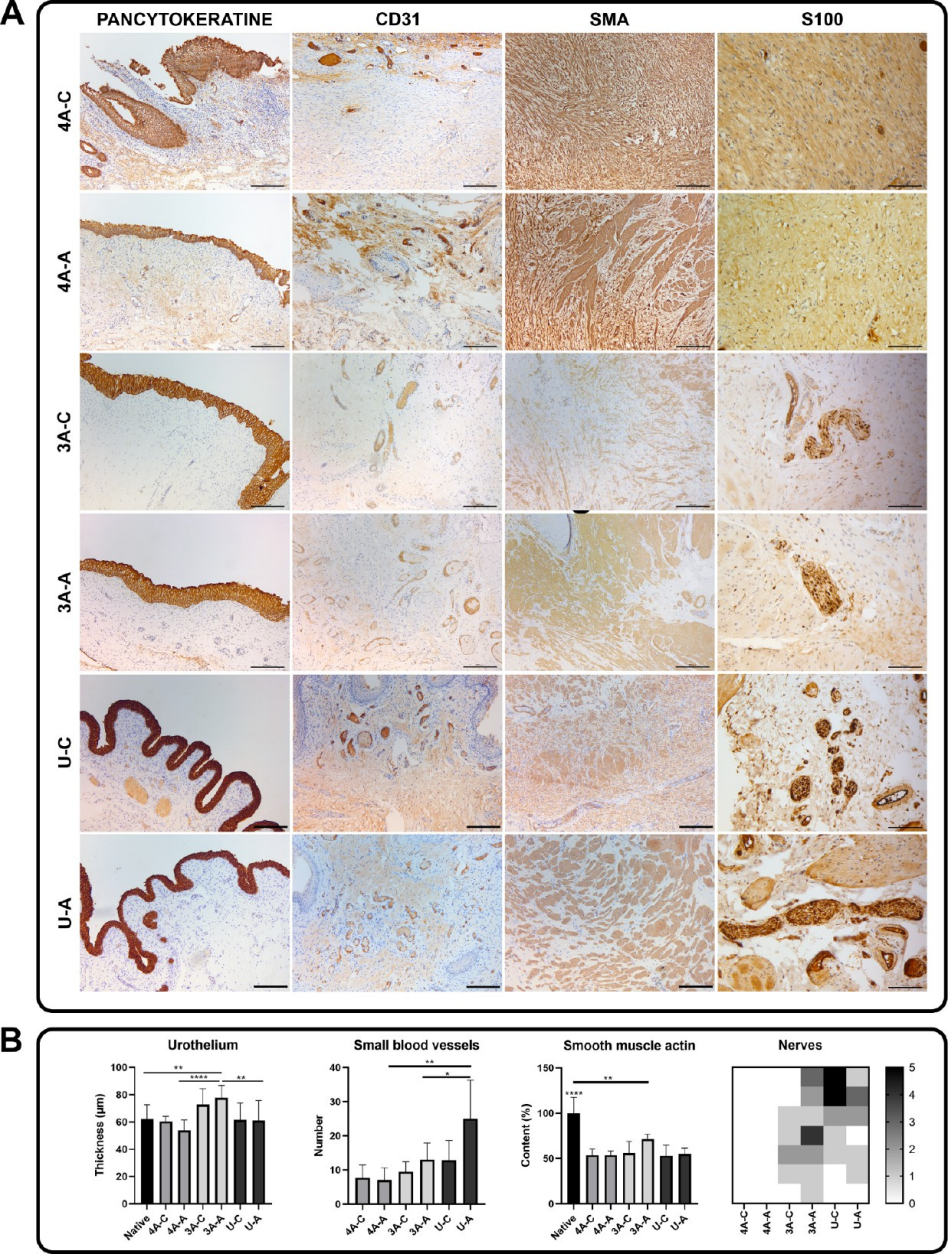
Evaluation of bladder wall regeneration. (A) Immunohistochemical analysis of urothelium layer (pancytokeratin), blood vessels (CD31), smooth muscle layer (SMA), and nerves (S100). Light microscope, scale bar = 200 μm, for S100 scale bar = 100 μm. (B) Numerical representation of immunohistochemical results. 4A—4-armed BAM-CC; 3A—3-armed BAM-CC; U—UROGRAFT; C—center; A—arm.

## Discussion

We developed a method of manufacture of a new tissue engineered graft (UROGRAFT) recommended for augmentation cystoplasty. The UROGRAFT is based on a composite of an extracellular matrix obtained by decellularization of porcine urinary bladders and cross-linked with collagen type I and dialdehyde carboxymethyl cellulose (BAM–CC). The unique shape of the UROGRAFT is designed to be easily implantable into the urinary bladder during augmentation cystoplasty without the necessity to create additional bladder tissue excision and to minimize scar formation in the central graft region.

Our goal was to manufacture a highly biocompatible, biodegradable, and impermeable for urine biomaterial with adequate mechanical properties allowing for bladder filling and emptying, which could be in the future available as both an acellular graft (biomedical device) or cell seeded tissue engineered product (combined ATMP-biomedical device).

In our previous preclinical studies on urinary bladder regeneration in a porcine model, we found that BAM is a promising biomaterial for the reconstruction of large urinary bladder wall defects. BAM implanted into the bladder was well tolerated, remodeled, and replaced by the host tissues.^17,18^ These results determined the selection of BAM as the basis for manufacturing a tissue engineered graft for future clinical applications. We used porcine urinary bladders for the manufacture of BAM due to greater availability and lower interindividual variability of animals compared to human donors. Decellularization aims to remove any cells and cellular debris, including xenogeneic or allogeneic antigens (a-Gal epitopes and MHC antigens) that can be recognized as foreign by the host and cause an adverse inflammatory response and immune mediated rejection. The main extracellular matrix (ECM) proteins are highly conserved between mammals and well tolerated by xenogeneic recipients; therefore ECM based scaffolds obtained from different species, including humans, pigs, cows, and horses, can be routinely used in clinical applications.

Numerous decellularization protocols have been developed, including physical, biological, and chemical methods. Dynamic decellularization techniques have shown significant advantages over static methods, particularly in the context of whole organ decellularization. Dynamic approaches, as demonstrated in studies like Consolo et al. (2016), Bolland et al. (2007), Rosario et al. (2008), Faulk et al. (2014), and Garriboli et al. (2022) allow for enhanced perfusion and uniform decellularization, which are critical for preserving the ECM integrity and biomechanical properties.^20–24^ Unlike static methods, dynamic protocols mimic physiological conditions, improving the retention of critical matrix components and facilitating recellularization, as highlighted in Badylak et al. (2011) and Crapo et al. (2011).^25,26^ For bladder applications, maintaining ECM elasticity and strength is vital, as shown by Bolland et al. (2007) and Dahms et al. (1998), making dynamic decellularization a superior choice.^21,27^ Moreover, the continuous flow systems in dynamic methods help prevent clogging and enhance the removal of cellular debris, providing scaffolds with better structural and functional compatibility for tissue regeneration.^28^ Therefore, dynamic decellularization emerges as the preferred approach for bladder tissue engineering, aligning with the goal of achieving biomimetic and functional tissue replacements.

The development and implementation of an automated system for dynamic bladder decellularization represent a significant advancement in tissue engineering. Initially, a simple homemade system composed of a plastic box and an aquarium pump was used to create a continuous flow for effective tissue decellularization. However, the transition to a fully automated system for dynamic decellularization, designed and manufactured by Zellwerk GmbH, ensured higher precision, reliability, and reproducibility required for BAM manufacture for clinical application. This automation eliminated inconsistencies and enhanced the overall efficiency of the process. The dynamic decellularization system employed a sophisticated configuration where urinary bladders were connected to allow a continuous flow of decellularizing solutions. The solutions entered the bladder via tubing-stabilized ureters and exited through a stabilized urethral outlet, ensuring uniform exposure of all anatomical structures. The stabilization ring and flow mechanics contributed to effective flushing and preservation of the ECM, which is critical for maintaining the bladder’s structural and functional integrity.

An effective decellularization protocol must balance the removal of cellular material to reduce immunogenicity while preserving the structural, biochemical, and mechanical integrity of the ECM, which is critical for scaffold functionality in tissue engineering.^26^ The primary objective of the initial phase of the study was to evaluate the efficacy of various urinary bladder decellularization protocols, including the use of Triton X-100, SDS, and combinations of the two, and to assess their subsequent capacity to ECM integrity, maintain low levels of cytotoxicity, and prepare biomaterial for regenerative applications. Of the methods evaluated, protocol III, employing a combination of Triton X-100 and SDS, was identified as the most effective, demonstrating significant advantages in terms of cell removal and DNA reduction while maintaining ECM preservation and high biocompatibility. The efficiency of the decellularization process is a key aspect determining the suitability of the resulting matrix for biomedical applications. The removal of cells and residual DNA minimizes the risk of a host immune response and allows the matrix to be successfully colonized by new cells after implantation.^29,30^

Our histological and molecular analyses showed that protocol III was most effective in achieving complete decellularization. The combination of SDS and Triton X-100 in protocol III from the present study aligns with findings by Yang et al. (2010), where a similar multistep approach effectively removed cellular materials while preserving essential ECM components such as sulfated glycosaminoglycans (GAGs), collagen, and bioactive factors like VEGF and PDGF-BB. Hematoxylin and eosin (HE) staining and 4′,6-diamidino-2-phenylindole (DAPI) staining confirmed that protocol III successfully removed all nuclei from the tissue, outperforming protocols I and II.^19^ Protocol I (Triton X-100) was the least effective, leaving a significant number of cell nuclei (86.20 ± 25.55), while protocol II (SDS) showed better decellularization efficiency 38.98 ± 12.61. Similar limitations of the Triton X-100 were noted by Simões et al. (2017) and Crapo et al. (2011), indicating that Triton X-100 is more effective at removing cytoplasm than cells and thus can lead to cellular residues being left behind.^26,31^ It was also observed that the easiest removal of cells was from the region I (RI), where the number of nuclei was − 59.8 ± 14.60 (protocol I), 24.73 ± 24.8 (protocol II) and the most difficult for region III – 110 ± 19.64 (protocol I), 48.73 ± 36.42 (protocol II). This phenomenon can be attributed, at least in part, to the fact that the decellularizing solution is being directed to the apex, and the solution tubing is located in region III, where the impact is minimized.

The residual DNA content of tissues processed using protocol III was found to be 9.72 ± 2.89 ng DNA/mg dry weight, which is significantly less than that of protocol II (35.07 ± 2.36 ng DNA/mg) and native bladder tissue (3813 ± 554.9 ng DNA/mg). This finding highlights that the synergistic action of Triton X-100 and SDS (protocol III) allows complete decellularization without leaving cellular fragments. A similar strategy was employed by Vishwakarma et al. (2020) and Yang et al. (2010), achieving near-complete DNA removal by sequential application of detergents.^19,32^ Furthermore, Crapo et al. (2011), Faulk et al. (2014) and Zhang et al. (2021) suggested that SDS removes cells more efficiently, while Triton X-100 may assist in preserving ECM structures, thereby explaining the efficacy of protocol III.^23,26,33^

The results obtained in this study meet the minimum requirements to define a tissue as decellularized according to Crapo et al. (2011), i.e., a DNA concentration of less than 50 ng dsDNA per mg ECM dry weight and the absence of visible nuclear material in tissue sections stained.^26^ This is evidenced by the use of 4’,6-diamidino-2-phenylindole (DAPI) and H&E staining or DNA quantification, which aligns with the findings reported by Yang et al. (2010).^19^ These findings are particularly noteworthy as they are consistent with the decellularization protocols that were selected as the basis for this study. These results are consistent with the threshold values assumed by Yiu et al. (2024) and Simões et al. (2017) that a 90% reduction in DNA levels is critical for reducing the immunogenic response in decellularized scaffolds used in tissue engineering.^31,34^ In addition, Keane et al. (2012) and Gilpin and Yang (2017) emphasized the importance of complete DNA removal to avoid inducing adverse host responses.^29,30^

It should be emphasized that every decellularization protocol will alter ECM composition and cause some degree of ultrastructure disruption. In this study, freeze–thaw cycles were used across protocols I–III to destroy cell membranes through intracellular ice crystal formation mechanically. This method has low cytotoxicity and minimal impact on ECM composition, though it can slightly disrupt microstructure. Its limited efficiency in cell removal necessitates combining it with chemical or biological methods.^35^ Chemical methods, including ionic (SDS) and nonionic (Triton X-100) detergents used in protocols I–III, dissolve cell membranes and disrupt lipid–protein interactions. SDS effectively removes cells but can damage ECM, while Triton X-100 better preserves ECM structure but has limited cell removal capability. Biological methods utilizing enzymes can degrade ECM ultrastructure and leave residues that may trigger immune responses.^33,36^ Scanning electron microscopy (SEM) and the quantification of extracellular matrix (ECM) proteins further highlighted the advantages of protocol III. SEM analysis demonstrated that collagen fibers remained intact and smooth, with pore sizes not exceeding 300 μm in decellularized tissue. This structural integrity is crucial for providing the mechanical and biochemical cues necessary for cell adhesion and proliferation in regenerative medicine. Quantification of ECM components showed that protocol III retained significantly higher concentrations of collagen, elastin, and laminin compared to protocol II (SDS) and native tissue. Collagen content was particularly enhanced, with protocol III yielding 642.3 ± 90.57 μg/mg, compared to 461.73 ± 22.23 μg/mg in protocol II and 207.99 ± 57.29 μg/mg in native tissue. The results for native tissue are lower than for manufactured BAMs because, for the same sample mass, the volume of native tissue is much smaller because it is occupied by cells. This does not mean that there is an increase in ECM protein content after decellularization, only that there is no excessive degradation. Similar trends were observed for elastin and laminin. These results suggest that protocol III not only ensures decellularization but also maintains the biochemical composition necessary for scaffold functionality. The findings indicate that protocol III not only ensures decellularization but also preserves the biochemical composition necessary for scaffold functionality. The study conducted by Faulk et al. (2014) revealed that SDS has the capacity to induce the fragmentation of collagen fibers and loss of laminin and elastin. However, the results of our study, in conjunction with those of Yang et al. (2010), demonstrate that the application of a multistep protocol (combination of SDS and Triton X-100) serves to minimize these negative effects and preserves the bioactive components of the ECM which are critical for cell proliferation and migration.^19,23^

One of the key aspects of assessing the extracellular matrix produced by decellularization is its potential cytotoxicity, resulting from detergent residues in the tissue. Biocompatibility tests using MTT assays demonstrated that BAMs manufactured using protocol III exhibited minimal cytotoxic effects on AD-MSCs at all concentrations tested (100% to 12.5%), with cell viability consistently exceeding 85%. This level of cell viability indicates that the material can be considered noncytotoxic, as the minimum viability, according to ISO 10993-12:2012, is 70%. This level of biocompatibility is crucial for subsequent successful cell repopulation and long-term implantation. In contrast, protocol II, although effective, showed reduced cell viability at higher concentrations, below 70%. This reduction in cell viability can probably be attributed to the presence of residual cytotoxic agents or suboptimal ECM behavior. Faulk et al. (2014) indicated that SDS can remain in the ECM and cause long-term cytotoxic effects, especially at high concentrations.^23^ Tissue washing after decellularization is a key step to remove residual detergents, such as Triton X-100 and SDS, which can be cytotoxic and adversely affect the biocompatibility of the resulting extracellular matrix (ECM). Faulk et al. (2014) analyzed the effects of different detergents on the basement membrane complex and highlighted the importance of thorough washing to minimize the potential for toxicity.^23^ Keane et al. (2012) and Gilpin and Yang (2017) emphasized the consequences of ineffective decellularization, including the presence of residual detergent residues that can lead to an adverse host response.^29,30^ Due to the use of different detergents (SDS and Triton X-100) in our study, it was decided to use up to 10 days of rinsing in PBS solution alternating with water to ensure even more effective detergent removal. The optimization of this step was crucial to obtaining a biocompatible matrix for tissue engineering and regenerative medicine applications. The absence of significant cytotoxicity in protocol III’s BAMs highlights its translational potential as a clinically viable material for regenerative applications. These findings align with those of Dhandapani and Vermette (2023), who reported that decellularized scaffolds must achieve both low cytotoxicity and high structural integrity to support functional cellular activities.^37^

Bladder acellular matrix (BAM) is usually cross-linked to improve its mechanical properties, such as tensile strength and resistance to degradation. This study compares several methods for cross-linking BAM with selected polymers (1% and 2% collagen, 1%, 5%, and 10% cellulose and collagen (1%)–cellulose (1%)). Importantly, in this study, BAM was cross-linked to reduce its porosity and permeability, which was a key objective in the graft manufacturing process. Type I collagen is the primary structural element of BAM, ensuring its integrity and functionality. At the same time, cellulose is known for its antimicrobial properties, making it an auspicious material for biomedical applications. Due to the intended clinical use, the cross-linking agents were clinical grade, ensuring their quality and safety. The following analyses were conducted to select the appropriate concentration of cross-linking agents. SEM analysis showed that BAM-collagen (2%) and BAM-cellulose (5%, 10%) composites had too large pores, which disqualified them for further testing. Therefore, BAM–collagen (1%), BAM–cellulose (1%), and BAM–collagen (1%)–cellulose (1%) composites were selected for further analysis. It was observed that the BAM–collagen (1%)–cellulose (1%) composite had an average of 39.123 μm, 115.163 μm, and 166.589 μm smaller pores compared to BAM, BAM–collagen (1%) composite and BAM–cellulose (1%) composite, respectively. Furthermore, it was observed that the BAM–collagen (1%)–cellulose (1%) composite maintained a relatively stable permeability for 24 h compared to BAM, which showed a marked decrease in permeability. Yang et al. used reduced graphene oxide (rGO) and polydopamine (PDA) to cross-link BAM. PDA was dissolved in Tris buffer (1.2 mg/mL, pH 9.5) and mixed with different concentrations of rGO (0.25, 0.5, and 1.0 mg/mL). They observed that the microstructure of the BAM composite did not change significantly after cross-linking, and the porosity of the BAM was retained.^38^ Zhao et al. used silk fibroin to cross-link BAM. They observed that the cross-linked scaffold had a porous structure with large pores of approximately 100 to 200 μm connected by a network of smaller pores.^39^ Cartwright et al. used hyaluronic acid (HA) at 0.05, 0.1, 0.2, and 0.5 mg/100 mL concentrations to cross-link BAM. The study assessed the permeability of BAM and cross-linked BAM using a 10 cm high water column, measuring the amount of water passed through the biomaterial per unit area over time. BAM was much more permeable (9.8 ± 1.6 cc/cm^2^ h) than cross-linked BAM (0.09 ± 0.02 cc/cm^2^ h). These results confirm that cross-linking with hyaluronic acid reduced the porosity of BAM.^40^

The BAM-collagen (1%)–cellulose (1%) composite degrades at 210 °C, which is slightly lower compared to BAM (223 °C). However, this difference does not affect the potential clinical application. Peng et al. assessed the degradation of cross-linked BAM by enzymatic degradation tests in the presence of type I collagenase. They observed that BAM cross-linked with 30 mg/mL dialdehyde carboxymethylcellulose had a significantly higher degradation resistance (50.83% weight loss in 24 h) compared to BAM (80.52% weight loss in 24 h).^41^ Yang et al. also used enzymatic degradation assays in the presence of type I collagenase to assess the degradation of cross-linked BAM. They observed that BAM degraded faster before cross-linking than BAM after cross-linking with reduced graphene oxide and polydopamine.^38^

Mechanical analysis showed that cross-linking BAM with collagen (1%) and cellulose (1%) significantly increases its stiffness, making it less susceptible to elastic deformation than BAM. The BAM-collagen (1%)–cellulose (1%) composite was found to be less elastic than natural bladder and BAM but still falls into the category of elastic materials due to its low Young’s modulus value. Similar results were obtained by Peng et al., who observed that cross-linking BAM with dialdehyde carboxymethylcellulose significantly increased the mechanical strength of BAM. Tests showed an increase in the stress at break and the modulus of elasticity, suggesting that cross-linking improves the material’s mechanical properties.^41^ Yang et al. also observed that cross-linking BAM with reduced graphene oxide and polydopamine improves its mechanical properties. Tensile tests showed an increase in tensile strength compared to BAM; electrochemical tests showed improved electrical conductivity of BAM after cross-linking.^38^ In summary, the BAM-collagen (1%)-cellulose (1%) composite met the cross-linking requirements, focusing on reducing porosity and permeability. Cross-linking also increased the mechanical properties of the BAM-collagen (1%)-cellulose (1%) composite; however, the composite still falls into the category of flexible materials.

Previously, we found that tissue regeneration in the bladder augmented with AD-MSCs seeded BAM is a result of the trophic effect of implanted cells, not their direct differentiation into bladder cells.^17^ Cells implanted on the scaffold die under the cytotoxic influence of urine and because of lack of oxygenation resulting from delayed revascularization at the site of implantation. Better results of regeneration observed in bladders reconstructed with cell-seeded scaffolds compared to acellular scaffolds are the result of the trophic influence of cells that serve as a feeder layer for native bladder cells migrating and colonizing scaffold. The efficacy and mode of migration are governed by a multifaceted set of biochemical and biophysical factors that are dependent on both cellular and scaffold properties. Based on the fact that native cell migration is critical for the regeneration of tissue-engineered bladders and knowing that urinary bladder smooth muscle cells have limited migration potential, we designed graft shape reducing the distance of migrating cells from graft edges to center and consequently minimizing the area of scar tissue formation in graft center.

Using a heat transfer model that mimics cell migration, we compared how the shape of the graft affects the rate of cell migration from the surrounding tissues to its center. We have found that different graft shapes with the same surface area may have different potential for cellularization and a tendency for scar formation in the graft center. Based on this analysis and surgical implantability of designed graft shapes into the bladder, first we selected 4-armed graft shape for preclinical testing of BAM-CC composite in urinary bladder augmentation in a porcine model.

We noticed already during the surgical procedure that the 4-armed BAM-CC composite graft is not suitable for bladder augmentation because, after implantation, it did not maintain its original shape. Our second choice was the 3-armed BAM-CC composite graft (with blunt arms), which was surgically implantable but the initial shape was not maintained in follow-up. Only the UROGRAFT (3-armed graft with pointed ends resembling a lily petal) kept its shape in all cases, which is why this shape was chosen for further analysis. In previous studies for bladder augmentation, we used BAM with the same graft area but in the shape of a circle.^17,18^ We observed that the regeneration of tissue layers was observed only on the edges of implanted material and gradually disappeared into the center, creating a scar. BAM-CC composite integrated well with native bladder tissue. Graft shape affected the rate of tissue remodeling and replacement with native tissue. Similar to our previous study, in the case of a 4-armed graft and of a 3-armed graft, scar tissue was formed in the central region. This was probably caused by the fact that after implantation, the graft shape changed to a rectangle or circle. In the UROGRAFT group, the scarring area was less noticeable and visible mainly from the inner side of the reconstructed bladder. Additionally, the regenerated muscle layer was more organized, also in the graft center. A better regeneration process was probably the effect of the shorter migration path of cells from native tissue to the graft center. The amount of regenerated muscle tissue was about half as much as in the control group. Additionally, a higher number of blood vessels developed, especially in the arm region of the UROGRAFT group. Innervation profile was similar in the 3-armed BAM-CC composite and the UROGRAFT groups, however in the finally selected shape, more S100 positive cells were observed in the graft center. This effect could be enhanced by seeding scaffolds with AD-MSCs, which, by the paracrine effect, could support tissue regeneration. Another conception is to use material divided into smaller fragments, which also could reduce the migration path to the graft center.^42^ The disadvantage of such a method is the need to perform several anastomoses on one bladder, which extends the time of the surgical procedure and could be the reason for complications during follow-up. Our solution enables the regeneration of bladder tissue using large grafts without the need to divide it into smaller parts. UROGRAFT implanted subcutaneously induced a slight inflammatory response, which could be the beginning of graft remodeling.^43^ Biocompatibility tests performed *in vivo* confirmed the lack of adverse effects induction by UROGRAFT. Taking into account all obtained results we can assume that our product is safe and can be evaluated in further studies.

## Conclusions

Summarizing, we have shown that the UROGRAFT developed in this study is a promising new product with the potential to be used in an augmentation cystoplasty. The technology of the UROGRAFT manufacture was planned from the beginning to be easily transferred to clinical practice. We used a dynamic automated system for tissue decellularization, clinical grade polymers for cross-linking, and a GMP bioreactor for cell seeding and culture. Preclinical studies on animal models revealed that the UROGRAFT is highly biocompatible and well tolerated by recipients. The method of porcine urinary bladder augmentation with the UROGRAFT is safe. The UROGRAFT showed promising potential to reconstruct clinically significant urinary bladder wall defects - regeneration of all urinary bladder wall tissue, including urothelium, smooth muscle, vessels, and nerves, was observed. Composite BAM-CC scaffolds provide an appropriate environment for AD-MSCs growth, therefore the UROGRAFT can be used in the future as an acellular graft (biomedical device) or cell-seeded tissue-engineered product (combined ATMP-biomedical device).

## Abbreviations

AD-MSCs: adipose-derived mesenchymal stromal cells
ATR-FTIR: attenuated total reflectance Fourier-transform infrared spectroscopy
BAM: bladder acellular matrix
BAM-CC: bladder acellular matrix–collagen (1%)–cellulose (1%)
b-FGF: basic fibroblast growth factor
DAPI: 4′,6-diamidino-2-phenylindole
DMEM/Ham’s F12: Dulbecco’s Modified Eagle Medium/Ham’s F12
DMSO: dimethyl sulfoxide
DSC: differential scanning calorimetry
ECM: extracellular matrix
EDTA: ethylenediaminetetraacetic acid
ELISA: enzyme-linked immunosorbent assay
FBS: fetal bovine serum
FEM: finite element method
GMP: good manufacturing practice
HA: hyaluronic acid
H&E: hematoxylin and eosin
ISO: International Organization for Standardization
KIU: kallikrein inhibitor units
PBS: phosphate-buffered saline
RT: room temperature
SDS: sodium dodecyl sulfate
SEM: scanning electron microscopy
SIS: small intestinal submucosa
VEGF: vascular endothelial growth factor
